# Investigation of somatic single nucleotide variations in human endogenous retrovirus elements and their potential association with cancer

**DOI:** 10.1371/journal.pone.0213770

**Published:** 2019-04-01

**Authors:** Ting-Chia Chang, Santosh Goud, John Torcivia-Rodriguez, Yu Hu, Qing Pan, Robel Kahsay, Jonas Blomberg, Raja Mazumder

**Affiliations:** 1 Department of Biochemistry & Molecular Medicine, George Washington University Medical Center, Washington, DC, United States of America; 2 The School of Systems Biology, George Mason University, Manassas, VA, United States of America; 3 Department of Statistics, The George Washington University, Washington, DC, United States of America; 4 Department of Medical Sciences, Uppsala University Hospital, Uppsala, Sweden; 5 McCormick Genomic and Proteomic Center, George Washington University, Washington, DC, United States of America; Plymouth University, UNITED KINGDOM

## Abstract

Human endogenous retroviruses (HERVs) have been investigated for potential links with human cancer. However, the distribution of somatic nucleotide variations in HERV elements has not been explored in detail. This study aims to identify HERV elements with an over-representation of somatic mutations (hot spots) in cancer patients. Four HERV elements with mutation hotspots were identified that overlap with exons of four human protein coding genes. These hotspots were identified based on the significant over-representation (p<8.62e-4) of non-synonymous single-nucleotide variations (nsSNVs). These genes are TNN (HERV-9/LTR12), OR4K15 (HERV-IP10F/LTR10F), ZNF99 (HERV-W/HERV17/LTR17), and KIR2DL1 (MST/MaLR). In an effort to identify mutations that effect survival, all nsSNVs were further evaluated and it was found that kidney cancer patients with mutation C2270G in ZNF99 have a significantly lower survival rate (hazard ratio = 2.6) compared to those without it. Among HERV elements in the human non-protein coding regions, we found 788 HERVs with significantly elevated numbers of somatic single-nucleotide variations (SNVs) (p<1.60e-5). From this category the top three HERV elements with significantly over-represented SNVs are HERV-H/LTR7, HERV-9/LTR12 and HERV-L/MLT2. Majority of the SNVs in these 788 HERV elements are located in three DNA functional groups: long non-coding RNAs (lncRNAs) (60%), introns (22.2%) and transcriptional factor binding sites (TFBS) (14.8%). This study provides a list of mutational hotspots in HERVs, which could potentially be used as biomarkers and therapeutic targets.

## Background

Endogenous retroviruses (ERVs) have been embedded in the primate genomes for over 30 million years [1, 2]. Typically, the genetic structure of ERVs contains the internal coding sequencing of the four proviral genes (gag, pro, pol and env) along with two long terminal repeats (LTRs) [3]. Over the course of time, most ERVs in the human genome have been severely damaged in their original genetic structure due to the accumulation of mutations, insertions, deletions and translocations that have spliced out the original coding region of proviral genes between two flanking LTRs [4, 5]. Solitary LTRs are the most common ERVs within the human genome [4, 5].

Human endogenous retroviruses (HERVs) account for approximately eight percent of the human genome [6] and have been classified into three main classes I, II and III. This classification is based on sequence similarity to different genera of infectious retroviruses [7]. Over 22 distinct HERV groups across three classes have been reported [8]. Gamma- and Epsilon-retrovirus like (GE; earlier called Class I) HERVs are linked to gamma-retroviruses like murine leukemia virus (MLV); It includes HERV-W (HERV17/LTR17/ERV-W), HERV-H (HERV-H/LTR7), and other subgroups. The Alpha and Beta-retrovirus like (AB; earlier called Class II) HERVs [9] are related to beta-retroviruses such as mouse mammary tumor virus and include several types of HERV-K (HML families) elements. Spumavirus like (S; earlier called Class III) HERVs are distantly related to spumaviruses and include HERV-L (HERV-L/MLT2) and HERV-S (HERV18/LTR18). Classifications of HERV elements are currently not entirely consistent due to varying approaches used to detect HERV sequences [8, 10]. This leads to a variable number of identified HERV groups based on the bioinformatic methodology and algorithm used and can cause inconsistencies in HERV classification [11, 12]. Recent work performed by Vargiu et al. has been able to systematically identify and classify 3,173 HERVs in the human genome [13], thereby providing some consistency in HERV classification. Of the 3,137 HERVs, 1,214 canonical (homogeneous) and 1,923 non-canonical (heterogenous) HERVs were separately placed into 39 and 31 groups (clades) respectively. This work builds upon a huge volume of previous work on repetitive elements and evolutionary analysis of ERVs [14, 15] [16–18].

Over the course of evolution, majority of HERVs within the human genome have gradually lost their original protein coding functions [19]. However, HERVs have been indirectly linked to the various diseases, including human preimplantation embryogenesis [20], multiple sclerosis[21], cancers [22–24] and neurological disorders [25]. In cancers, for example, Np9, which is encoded by HERV-K (HML groups) elements, has been proposed to be involved in oncogenomic mechanisms through the LNX/Numb/Notch pathway [26, 27]. HERV LTRs have been reported to participate in human tumorigenesis by regulating the expression of its adjacent genes [28, 29]. For example, a mutation found in a HERV LTR leads to the activation of syncytin-1 encoded by HERV-W Env with high expression in bladder carcinoma [30]. It has been proposed that somatic mutations are associated with aberrant activation of stem cell-associated retroviruses (SCAR) and with stem cell-like phenotypes of cancer cells, clinical intractability of human malignancies, and increased likelihood of therapy failure and death from cancer [31].

Next-Generation Sequencing (NGS) has immensely aided the identification of genetic variations and their role in human diseases [32–34]. The availability of single nucleotide variation (SNV) databases and locus specific disease-related annotation databases has helped researchers map mutations to potential biomarkers [35]. In this study, we have analyzed such datasets to explore pan-cancer mutations in HERV elements. Although, it has been shown in several studies that such pan-cancer analysis can help identify patterns of driver mutations [36–38], to the best of our knowledge no such study has been performed on HERV elements.

This study explores correlations between HERV elements and cancers by identifying somatic mutation hotspots in the human genome, followed by a detailed review of functional annotations available for these genomic regions.

## Material and methods

### Data integration

#### SNV data

All SNV data was retrieved from BioMuta. BioMuta v3.0 [35, 39] is a comprehensive non-redundant data set of SNVs found in cancer within the coding region of hg19/GRCh37 compiled from multiple sources, including The Cancer Genome Atlas (TCGA) [40], CGHub data portal (https://cghub.ucsc.edu/), ClinVar [41], Catalog Somatic Mutations in Cancer (COSMIC) [42], International Cancer Genome Consortium (ICGC) [43], Integrative Onco Genomics (IntOGen) [44], UniProtKB [45], literature mining, and manual curation. For this study, BioMuta v3.0 was expanded to include SNVs in the non-coding regions of the human genome (hg19/GRCh37). The non-coding SNVs mutation data was extracted from (ICGC) (Version 23) [43] and COSMIC (version 79) [42]. This non-coding database was derived from whole genome sequencing (WGS) data and was restricted to regions excluding coding domain sequences based on the annotations available through UCSC genome browser (https://genome.ucsc.edu/). It is important to note that single nucleotide polymorphisms (SNPs) were filtered out for both coding and non-coding SNV datasets by Mutect 2 for TCGA [46] and by MuTect and Strelka [47] for ICGC [48] and DNA-Seq analysis pipelines for TCGA is listed in (https://docs.gdc.cancer.gov/Data/Bioinformatics_Pipelines/).SNV identification in repetitive regions is handled based on published methods [49–51].

#### HERV data

Comprehensive HERV data set based on hg19/GRCh37 (S1 Table) was obtained using methods described by Vargiu et al. [13]. The detection of HERV elements is based on three basic principles: (a) detection of candidate LTRs, (b) detection of chains of conserved retroviral motifs fulfilling distance constraints, and (c) an attempt at reconstruction of original retroviral protein sequences, combining alignment, codon statistics and properties of protein ends. All classifications and nomenclature of HERV elements that is used for this study is available in S2 Table. The term canonical HERV is defined to be a HERV sequence that comes from one HERV group [13]. Otherwise, a HERV element is considered non-canonical if composed of two or more HERV groups. The names of groups are based on prior usage in the literature and from RepeatMasker [16].

### DNA functional elements data

The DNA functional elements data set for protein non-coding regions is comprised of: tRNA (version 1.23) [52–54]; CpG island (download date: Oct. 2015) (UCSC Genome Browser); Open Regulatory Annotations (version 3.0) [55, 56]; microRNA sites and their target sites (version 7.1) [57]; pseudo exons (release 60) [58, 59]; VISTA enhancers (download date: Oct. 2015) [60], HAca box elements; CDBox elements; SnoRNA/ScaRNA sites (version 3) [61], alternative splice sites (download date: Oct. 2015) (UCSC genome Browser); transcription factor binding sites (version 3.0) [62, 63], introns (download date: Oct. 2015) (UCSC genome Browser), and long non-coding RNAs (v4.0) [64].

### Over-representation analysis

SNVs were mapped to HERV elements based on their genomic coordinates. SNVs could be either from human protein coding region or protein non-coding region. Retrovirus coding region such as gag, pro, pol and env are not considered as human protein coding region unless there is annotation in UniProtKB/Swiss-Prot [45] that indicates that they are indeed part of a protein coding sequence. The Binomial test [65, 66] was then used to evaluate the significance of the over-represented SNVs in each HERV element, by comparing its observed SNV number to the expected SNV number in each HERV element on human protein coding region or non-coding region, respectively [67, 68]. The calculation of the expected number n(E) of SNV sites in each HERV element is expressed as follows:

$$p(F)=\frac{n(F)}{L}$$

Where n(F) is the total number of nucleotides at each HERV element (total base pairs of each HERV element) and L is the total number of nucleotides of the genome (total base pairs of human chromosomal protein coding or non-coding region). The probability p(F) of observing a nucleotide from the human genome at a certain HERV element is calculated by taking the value of n(F) divided by total length of human chromosomal protein coding or protein non-coding genome L.

$$n(E)=N\times p(F)=N\times \frac{n(F)}{L}$$

Here, N is the total number of variation sites found in the human protein coding or non-coding part of the genome. Assuming that somatic SNV sites are equally likely to be found along the entire genome, the value of n(E) gives us the expected number of SNV sites that would be found in the HERV regions of the genome. The expected ratio in whole human genome is 0.019 (total number of SNVs in coding and non-coding region divided by total number of bases in the human genome). To evaluate the expected ratio in the whole genome, random sampling of permutation [69] was performed in R (http://www.R-project.org/) for comparing the observed ratio in random fragments and calculating the number of SNVs in each fragment (1000 bases in one fragment). (S3 Table)

Binomial statistic was used to calculate the p-value of the expected versus observed as follows:

$$P-value=\sum_{n=n(O)}^{N}\left(\frac{N!}{n!(N-n)!}\right)\times P{\left(F\right)}^{n}\times {\left(1-p(F)\right)}^{N-n}$$

Where n(O) is the observed number of SNV sites within the HERV elements being examined. Bonferroni correction was used to calculate the threshold for the p-Value (0.05/n) (n represents the number of Binomial tests we performed here). The p-value used as our significance cutoff was 8.62e-4 (0.05/58) for the protein coding region nsSNVs (S4 Table). For protein non-coding region SNVs in HERVs, the significance cutoff was 1.60e-5 (0.05/3,130) (S5 Table).

### Differential expression analysis

BioXpress is a gene expression database, which provides differential expression of both gene and miRNA in cancer [39, 70]. With respect to a specific cancer type, differential expression analysis using DESeq2 [71] is performed on expression levels of each gene or miRNA in tumor and adjacent non-tumor samples. Current version of BioXpress includes 34 TCGA cancer types (mapped to 73 DOIDs (Cancer Disease Ontology IDs)) [72], in which 20,502 genes and 1,965 miRNAs were analyzed and 18,846 genes and 710 miRNAs have been observed to be significantly differentially expressed in at least one cancer type. Genes of interest (HERVs that overlap with protein coding genes and are somatic mutation hotspots), identified in this study, were further explored in BioXpress to find out if they are significantly overexpressed in cancer.

### Survival analysis

Key nsSNVs that were identified were further investigated by log-rank test to evaluate their possible impact on patient survival. Patient clinical information was retrieved from TCGA (https://portal.gdc.cancer.gov/). Each key nsSNV was retrieved which have significant (p < 0.05) differential expression in the certain cancer types. BioMuta nsSNVs from non-TCGA sources was removed since BioXpress only uses TCGA data. Cancer patients were divided into two groups: one group where the patients have the key nsSNV and the other where they do not. Log-rank test was applied to test the death time distributions for the two groups. The Cox model was used to adjust for factors such as age at initial diagnosis, pathological stage and gender. SAS (version 9.3) using previously published method was used to perform the analysis [73].

## Results

### Genome-wide identification of somatic SNVs in HERV elements

In the protein coding region of the human genome 2,867,887 sites impacted by somatic nsSNVs were found. Whereas, in human protein non-coding region, 59,205,289 SNV sites with somatic SNVs were identified (S6 and S7 Tables). To confirm the coverage of SNVs in whole human genome, all SNVs were mapped to the genome. S1 File shows the distributions of all SNVs in chromosome 1 to 22, X, Y. The gap in the plot represents the repeat sequence and low complexity centromeric region where SNVs are hard to identify.

A total of 2,543 somatic nsSNVs were identified in 25 HERV groups that overlap with human protein coding regions (S1 and S6 Tables). Amongst them, 919 nsSNVs were identified in seven Gamma-retrovirus/Epsilon-retrovirus-related (GE) canonical HERV groups. The rest 1,624 nsSNVs were found in 20 non-canonical HERV groups. Ten of the groups were from Gamma-retrovirus /Epsilon-retrovirus-related (GE) non-canonical retrovirus, which involved 50.1% of the total examined nsSNVs. Five out of 20 groups belonging to Alpha-retrovirus/Beta-retrovirus-related (AB) retroviruses contained 23.5% of the nsSNVs. 20.4% of nsSNVs were within two Spumavirus related (S) HERV groups. 5.8% of the nsSNVs were found in the “Uncertain Errantivirus‑like” group and two unclassified HERV groups.

To investigate the distribution of somatic SNVs in HERV elements in protein non-coding regions, we mapped SNVs present in protein non-coding genomic regions HERV genomic coordinates. The results indicate 433,409 human chromosomal non-coding SNVs are located in HERV elements. 167,561 of them (38.7%) were in canonical HERV groups (Table 1 and S7 Table). Of these 167,561 mutations, 135,032, or 80.8% were found within HERV Gamma-retrovirus/Epsilon-retrovirus-related (GE) retroviruses. 18,295 (10.9%), and 14,243 (8.3%) were found in HERV Alpha-retrovirus/Beta-retrovirus-related (AB) retroviruses, and Spumavirus-related (S) retroviruses, respectively. The rest 265,848 SNVs were found in the non-canonical HERV groups. The non-canonical group classifications are Gamma-retrovirus/Epsilon-retrovirus-related (GE) retroviruses, Alpha-retrovirus/Beta-retrovirus-related (AB) retroviruses, Spumavirus-related (S) retroviruses, Uncertain Errantivirus‑like proviruses, and unclassified groups. The proportion of mutations found in these groups are 72.6% (193,268/265,848), 18.5% (49,324/265,848), 8.3% (22,043/265,848), < 0.001% (89/265,848), and 0.4% (1,124/265,848) respectively (S7 Table).

**Table 1:** Human whole genome SNVs in HERV elements.

**Protein Coding region / HERVs**								
**HERV sequence**	**Canonical**	**Noncanonical**						
**HERV supergroups**	GE^a^	GE	AB^b^	S^c^		Uncertain Errantivirus-like proviruses		Unclassified
**Total No. of nsSNVs**	919	1,624						
**Percentage of nsSNVs**	100%	50.10%	23.50%	20.40%		5.80%		0.20%
**Non-coding region / HERVs**								
**HERV sequence**	**Canonical**			**Noncanonical**				
**HERV supergroups**	GE	AB	S	GE	AB	S	Uncertain Errantivirus-like proviruses	Unclassified
**Total No. of nsSNVs**	167,561			265,848				
**Percentage of nsSNVs**	80.80%	10.90%	8.30%	72.60%	18.50%	8.30%	< 0.001%	0.40%

^a^ Gamma-retrovirus/Epsilon-retrovirus-related

^b^ Alpha-retrovirus/ Beta-retrovirus-related

^c^ Spumavirus-related

### Somatic SNV hotspots in HERV elements

To understand whether certain HERV elements contain a significantly different number of SNVs than expected, we compared the observed mutations within each HERV element to expected number of mutations.

#### SNVs from human protein coding region with HERV elements

**Fig 1:**
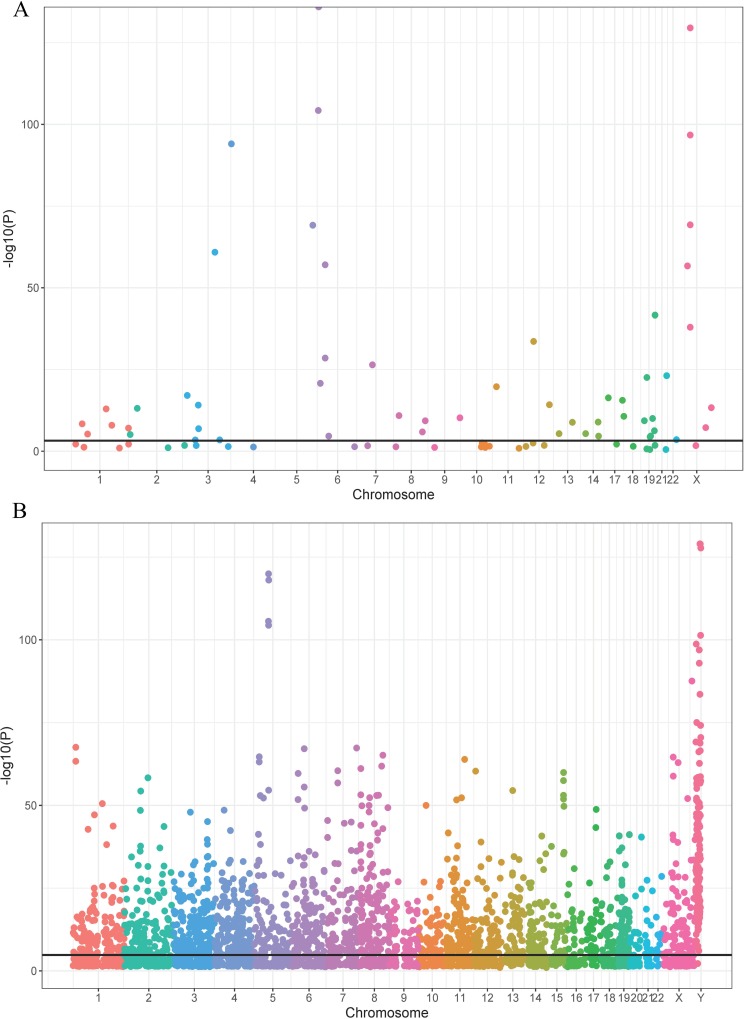
SNVs within HERV elements on human protein coding and protein non-coding regions. (A) nsSNVs in HERV elements on the human protein coding region by chromosome. The X-axis represents chromosomes while the Y-axis shows the transformed (-log10[P]) P-value. The transformed P-value was used to obtain the specific HERV elements with significant representation of nsSNVs. The threshold is 4.06 (-Log10 (8.62e-4)). Each dot on the Manhattan plot represent one HERV element; the different colors interprets the HERV element in a different chromosome. If the HERV element contains significantly more nsSNVs than expected nsSNVs, the HERV element is interpreted with a significant over-representation of nsSNVs. Approximately 42% HERV elements have a significant over-representation of nsSNVs. (B) SNVs in HERV elements on human non-coding region by chromosome. The threshold is 4.80 (-Log10 (1.60e-5)).

There are several HERV elements that overlap with the exon of genes. We identified HERVs which contained significantly more nsSNVs than expected (p < 8.62e-4). There were 83 HERVs with 2,543 nsSNVs (Fig 1A). 39% of the elements (32/83), encompassing 1,855 of the 2,543 nsSNVs, have significant numbers of nsSNVs (S4 Table). Majority of the HERV elements (28/32, 87.5%) are in 18 different HERV groups, and of these, 28 HERVs have significantly fewer nsSNVs than expected in the protein coding region. The remaining 4 (12.5%) HERV elements include significant over-representation of nsSNVs (Table 2 and S8 Table) containing 492 of the 1,855 (26%) and hence was prioritized for further functional analysis. In total, 492 nsSNVs are concentrated in the exon regions of 4 genes: tenascin N (TNN) (with HERV-9/LTR12) (p = 6.67e-4), zinc finger protein 99 (ZNF99) (with HERV-W/HERV17/LTR17) (2.63e-26), killer cell immunoglobulin like receptor, two Ig domains and long cytoplasmic tail 1 (KIR2DL1) (with MaLR/MST) (3.26e-7), and olfactory receptor family 4 subfamily K member 15 (OR4K15) (with HERV-IP10F/LTR10F) (2.58e-5) (Table 2, S8 and S9 Tables).

**Table 2:** Four HERV elements with significantly over-represented nsSNVs mapped to four genes.

HERV Sequence	HERV ID	HERV groups	Gene	Chromosome	Length	Mutations	Expected mutations	Difference	P-value
**Non-canon**	6114	HERV-9/ LTR12	TNN	1	264	35	20.47	14.52	6.67e-04
**Non-canon**	4780	MST/ MaLR	KIR2DL1	19	702	92	54.43	37.56	3.26e-07
**Non-canon**	4062	HERV-IP10F/ LTR10F	OR4K15	14	1044	116	80.95	35.04	2.58e-05
**Non-canon**	4673	HERV-W/ LTR17/ HERV17	ZNF99	19	1585	249	122.91	126.08	2.63e-26

#### SNVs from human protein non-coding regions mapping to HERV elements

There are 1,820 HERV elements in protein non-coding regions which contain significant over- or under-representation of SNVs (Fig 1B and S5 Table) (p < 1.60e-5). Most of the HERV elements (1,032 or 57%) have significantly fewer mutations than expected. The remaining 788 HERV elements (43%) have an over-representation of SNVs, which account for more than 73% of the total SNVs. 193,439 SNVs (S5 and S10 Tables) were identified in 788 HERV elements that contained a significantly high number of mutations (p < 1.60e-5). The proportion of somatic mutations in canonical HERVs (41.7%) is lower than that of non-canonical HERVs (58.3%). For canonical HERVs, the proportion of somatic mutations in Gamma-retrovirus/Epsilon-retrovirus-related (GE) retroviruses, Alpha-retrovirus/Beta-retrovirus-related (AB) retroviruses, Spumavirus-related (S) retroviruses HERV groups are 80.9%, 9.3%, 9.8%, respectively. For non-canonical HERVs, the proportion of mutations in classes Gamma-retrovirus/Epsilon-retrovirus-related (GE) retroviruses, Alpha-retrovirus/Beta-retrovirus-related (AB) retroviruses, Spumavirus-related (S) retroviruses are 72.9%, 17.3%, and 9.8% respectively.

### Genome-wide pan-cancer analysis

#### HERV elements with over-representation of nsSNVs

**Fig 2:**
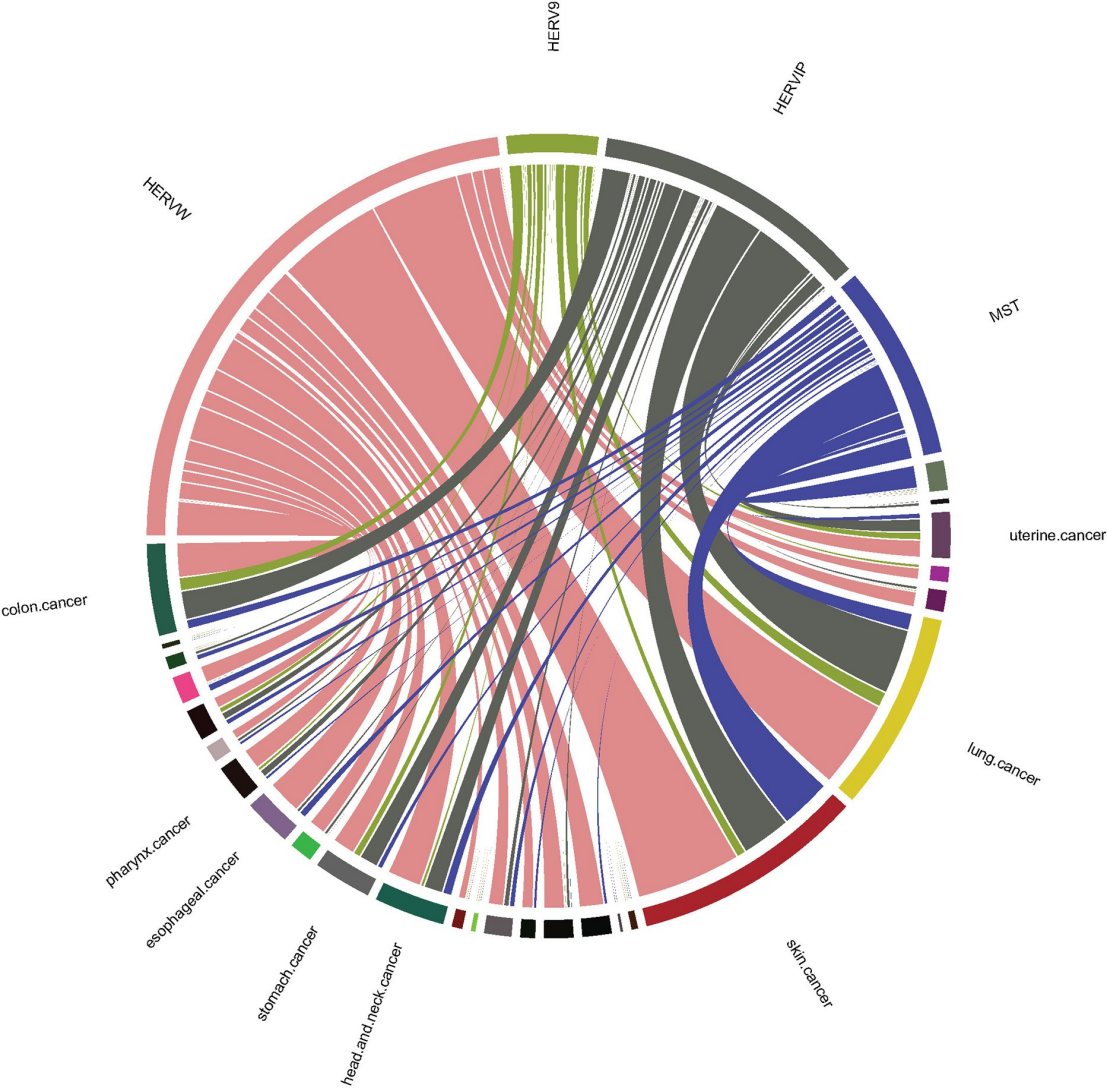
The relationship between HERV elements on the human protein coding regions with over-represented nsSNVs and multiple cancers. The CIRCOS plot represents the number of nsSNVs in each HERV element that are found in multiple cancer types. The Gamma-retrovirus/Epsilon-retrovirus-related non-canonical HERV elements which contain HERV-W/LTR17/HERV17, HERV-IP10F/LTR10F, and HERV-9/LTR12 include 81.6% nsSNVs. The Gamma-retrovirus/Epsilon-retrovirus-related non-canonical HERV elements are associated with top cancer types including skin cancer, lung cancer, and head & neck cancer. The proportion of nsSNVs in MST/MaLR, Spumavirus-related non-canonical HERV elements, is close to 18%, which associated with top cancer types including skin cancer, thyroid cancer, and lung cancer. Please note that all HERVs nomenclatures are presented by supergroups and see their relative/similar element in S2 Table.

To further investigate the impact of nsSNVs in HERV regions in different cancer types, cancer terms were unified using cancer disease ontology (DO) [72]. The relationship between nsSNVs in the protein coding region HERV elements and multiple cancer types is shown in Fig 2. It can be seen from Table 2 that 81.3% of the nsSNVs are from Gamma-retrovirus/Epsilon-retrovirus-related (GE) non-canonical HERV elements (HERV-W/LTR17/HERV17, HERV-9/LTR12 and HERV-IP10F/LTR10F). These nsSNVs are associated with at least 20 cancer types. The top three cancer types—skin cancer, lung cancer, and colon cancer—are associated with mutated sites in HERV-W/LTR17/HERV17. The proportion of mutations in HERV-W/LTR17/HERV17 was 50.6% (249/492). HERV element in Spumavirus-related (S) (MaLR/MST) is found in several cancer types. The proportion of mutations in (MaLR/MST) was 18.7% (92/492). This set of MaLR/MST mutations is found in many cancer types including skin cancer, thyroid cancer, and lung cancer.

#### HERV elements with an over-representation of SNVs from protein non-coding regions

SNVs in the human chromosomal non-coding region of HERV elements could also lead to carcinogenesis if they impact regulatory regions or protein binding sites [74]. Liang et. al. suggested the HERV elements with unstable genomic variants near lncRNA can trigger onset of human adenocarcinoma [75]. Based on our results (Fig 1B), we identified 788 HERV elements with significant over-representation of SNVs. S1 Fig indicates each of 788 HERV elements contains 144 SNVs on average, which are coming from skin cancer patient samples. The second highest number (29 SNVs on average) is from esophageal cancer. And the third highest number is from liver cancer (15 SNVs on average).

**Fig 3:**
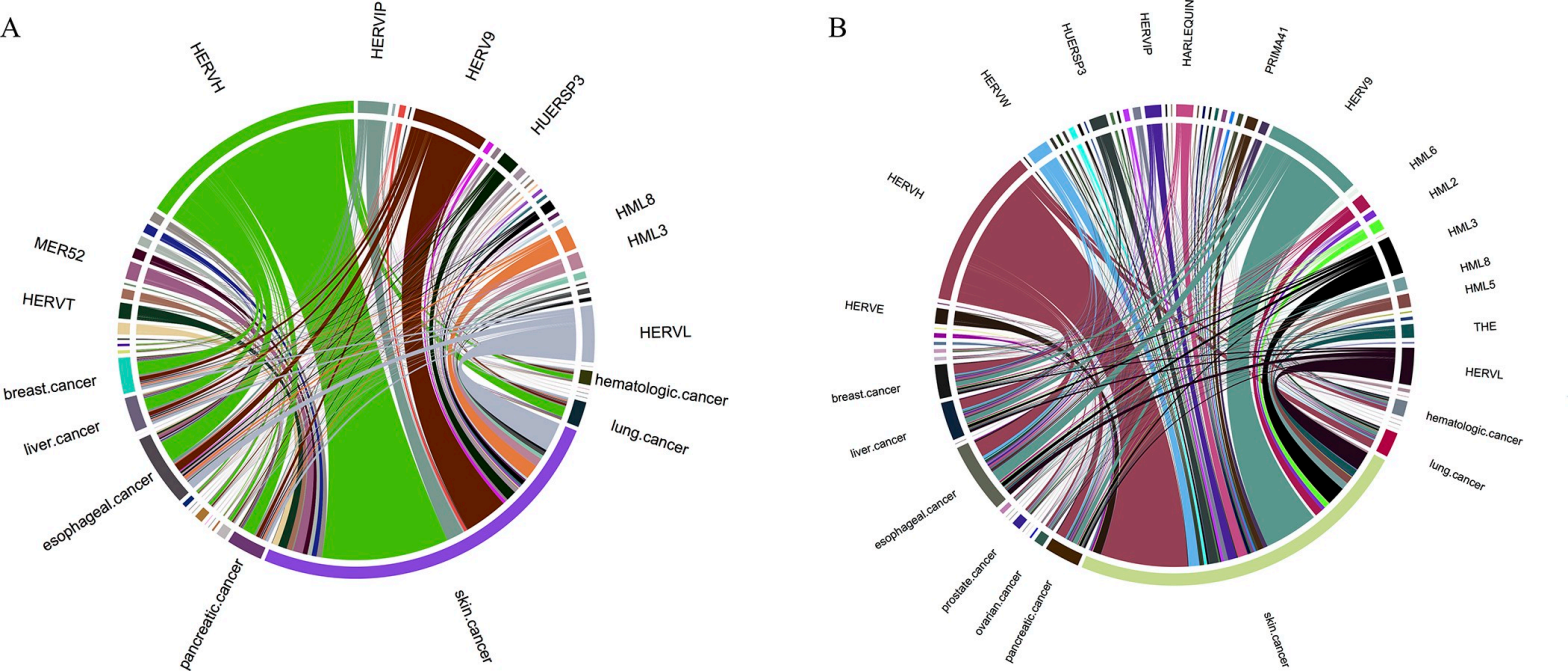
Canonical and non-canonical HERVs on human non-coding region and their relationships with cancer. (A) the relationship between 357 canonical HERV element on human non-coding region and cancers. The CIRCOS plot shows the number of SNVs in each canonical HERV element within human non-coding region and how they are associated with multiple cancer types. The proportion of Gamma-retrovirus/Epsilon-retrovirus-related canonical HERV elements which includes HERV-H/LTR7, HERV-9/LTR12, HERV-IP10F/LTR10F and HUERSP/MER52/LTR25 is close 60%. They are associated with multiple cancer types including skin cancer, esophageal cancer, and breast cancer. The proportion of Alpha-retrovirus/ Beta-retrovirus-related canonical HERV elements which includes HML-8/HERVK11I/MER11A is close 42%. They are associated with multiple cancer types including skin cancer. The proportion of Spumavirus-related canonical HERV elements which includes HERV-L/MLT2 is close 93.7%. They are associated with multiple cancer types including skin cancer. (B) the relationship between 431 non-canonical HERV element on human non-coding region and cancers. The CIRCOS plot shows the number of SNVs in each non-canonical HERV element within non-coding region and how they are associated with multiple cancer types. The proportion of Gamma-retrovirus/Epsilon-retrovirus-related non-canonical HERV elements which includes HERVH/LTR7, HERV9/LTR12 and HERV-IP10F/LTR10F is close 63% and their association with multiple cancer types including skin cancer, esophageal cancer, and breast cancer. The proportion of Alpha-retrovirus/ Beta-retrovirus-related canonical HERV elements which includes HML-3/HERVK9I/MER9 is close to 37%. The proportion of Spumavirus-related canonical HERV elements which includes HERV-L/MLT2 is close to 62.7%. They are found in multiple cancer types including skin cancer. Please note that all HERVs nomenclatures are presented by supergroups and see their relative/similar element in S2 Table and that three HERVs minor supergroups (MER52, THE, and HARLEQUIN) which present in the Fig 3 are listed in S2 Table.

Fig 3 shows the distribution of SNVs in 357 canonical and 431 non-canonical HERV elements that map to protein non-coding regions. In both canonical (Fig 3A) and non-canonical (Fig 3B) Gamma-retrovirus/Epsilon-retrovirus-related (GE) HERVs, HERV-H/LTR7 and HERV-9/LTR12 contain the largest numbers of SNVs from skin cancer, esophageal cancer, and breast cancer. In canonical Alpha-retrovirus/Beta-retrovirus-related (AB) HERVs, HML-8/HERV-K11I/MER11A contain the most somatic mutations. HML-3/HERV-K9I/MER9 is the most affected non-canonical Alpha-retrovirus/Beta-retrovirus-related (AB) HERVs. In both canonical and non-canonical Spumavirus-related (S) HERVs, HERV-L/LTR7 has the highest SNV sites.

### Functional analyses

#### Expression of genes containing HERV element with over-represented nsSNVs

Even though the nsSNVs in our dataset are somatic mutations, the correlation between gene expression, HERVs, and each cancer type can provide insights into the role of these genes in cancer. Our results show that nsSNVs found in multiple cancer types are located within exomes of *TNN*, *KIR2DL1*, *OR4K15*, and *ZNF99* genes. We examined whether or not their differential expression were significant in 34 cancer types.

**Fig 4:**
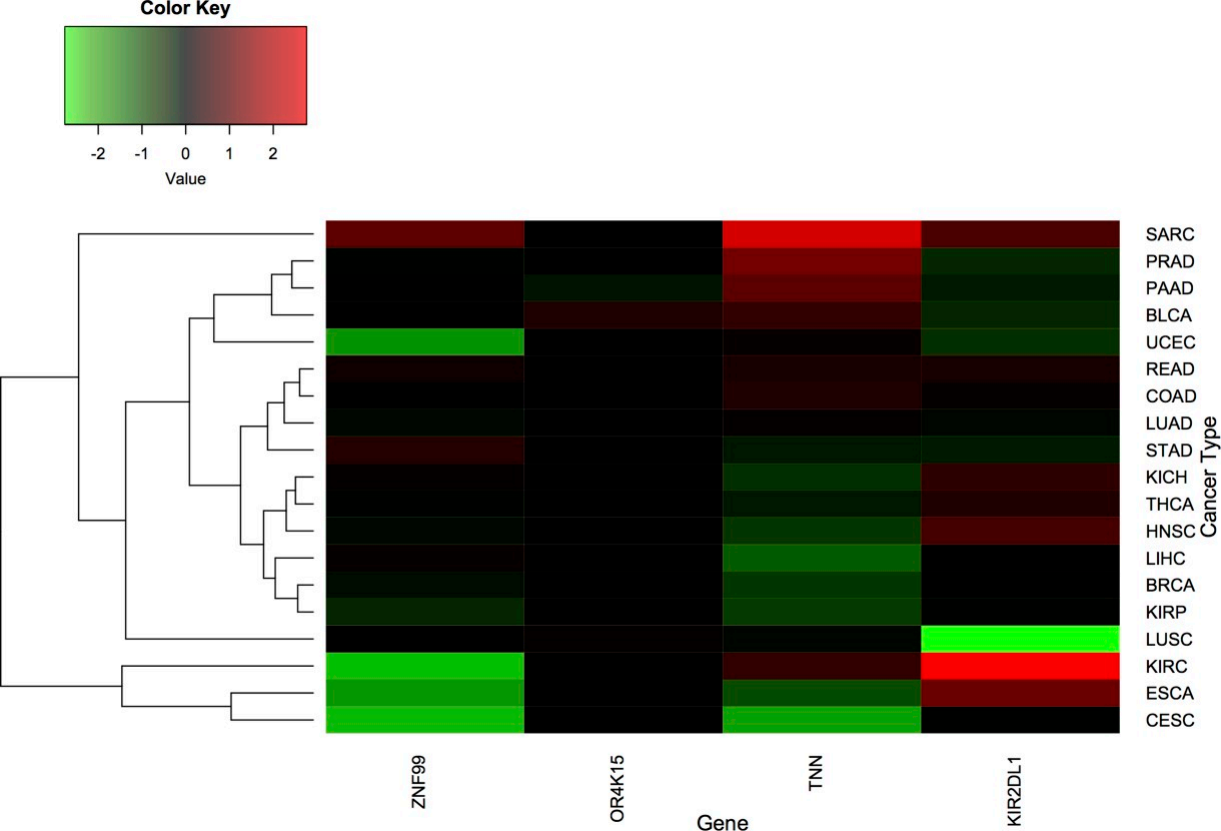
The differential expression of four key genes identified in this study across multiple cancers. X-axis is the gene name from left to right (ZNF99, OR4K15, TNN, KIR2DL1). Y-axis is cancer type. Cancer types are as reported by TCGA from top to bottom: SARC (Sarcoma), PRAD (Prostate adenocarcinoma), PAAD (Pancreatic adenocarcinoma), BLCA (Bladder Urothelial Carcinoma), UCEC (Uterine Corpus Endometrial Carcinoma), READ (Rectum adenocarcinoma), COAD (Colon adenocarcinoma), LUAD (Lung adenocarcinoma), STAD (Stomach adenocarcinoma), KICH (Kidney Chromophobe), THCA (Thyroid carcinoma), HNSC (Head and Neck squamous cell carcinoma), LIHC (Liver hepatocellular carcinoma), BRCA (Breast invasive carcinoma), KIRP (Kidney renal papillary cell carcinoma), LUSC (Lung squamous cell carcinoma), KIRC (Kidney renal clear cell carcinoma), ESCA (Esophageal carcinoma), and CESC (Cervical squamous cell carcinoma and endocervical adenocarcinoma). The heatmap represents the differential expressions across multiple cancers. Red represents the level of over-expression of the gene. Green represents the level of under-expression of the gene. The value of differential expression was calculated by Log2Fold-Change.

We found that *TNN* was significantly over-expressed in kidney renal clear cell carcinoma (KIRC) (p = 0.024) and prostate cancer (p ≈0), but significantly under-expressed in liver (p = 0.003) and breast cancer (p = 0.003). 68% (49/72) of patients with KIRC and 84.6% (44/52) of patients with prostate cancer have the same trend of over-expressed *TNN* (Fig 4). Conversely, 88% (44/50) of patients with liver cancer and 54.4% (62/114) of patients with breast cancer have a similar tendency of under-expressed *TNN*. Additionally, *KIR2DL1* was significantly over-expressed in kidney cancer (p ≈0) while significantly under-expressed in lung cancer (p ≈0). 91.7% (66/72) patients had a positive correlation of up-regulated *KIR2DL1* in kidney cancer. 94.1% (48/51) patients had a direct correlation of down-regulated *KIR2DL1* in lung cancer (Fig 4). Moreover, *ZNF99* was significantly under-expressed in kidney cancer (p ≈0). 84.72% (61/72) of patients with kidney have the same trend of under-expression of *ZNF99* in kidney cancer (Fig 4).

#### SNVs impact on HERV region and protein functional sites

In addition to our gene expression analysis, we counted the numbers of nsSNVs that may have an impact on functional sites within these four genes. S11 Table provides the total number of nsSNVs affecting post-translational modification of amino acids. In the gene *TNN*, cancer-associated nsSNVs within HERV elements were found to likely impact amino acid post-translational modification (PTM) sites that led to phosphorylation gain or loss. In *KIR2DL1*, mutations were found to likely impact multiple functions such as phosphorylation, glycosylation, and ligand binding site. In *OR4K15* and *ZNF99*, nsSNVs affect the modification of amino acid phosphorylation and glycosylation.

We combined the results of nsSNVs found in cancers with significant differential gene expression data and impacted PTM sites (Table 3). Three nsSNVs in TNN are found in kidney cancer and breast cancer samples. One of these three nsSNVs impacts a PTM site of the protein. 12 nsSNVs in *KIR2DL1* are found in lung cancer and kidney cancer samples. Six out of these 12 nsSNVs affect PTM sites on this protein. In *ZNF99*, seven nsSNVs are found in kidney cancer and there are no PTM site affecting nsSNVs.

**Table 3:** 22 nsSNVs in the four significantly differentially expressed of genes and their functional sites.

Gene	Chromo some	Position_ N^a^	Ref_ N^b^	Var_ N^c^	Position_ A^d^	Ref_ A^e^	Var_ A^f^	Predicted impact on function	DOID /Cancer type	HERV _Id	HERV group	HERV Supergroup
**TNN**	1	2061	C	A	687	H	Q	-	DOID:1612 / breast cancer	6114	HERV-9/ LTR12	GE^g^
**TNN**	1	2062	G	A	688	V	M	-	DOID:1612 / breast cancer	6114	HERV-9/ LTR12	GE
**TNN**	1	1991	T	C	664	V	A	Gain| Phosphorylation	DOID:263 / kidney cancer	6114	HERV-9/ LTR12	GE
**KIR2DL1**	19	64	C	A	22	H	N	Gain| Phosphorylation	DOID:1324 / lung cancer	4780	MST/ MaLR	S^h^
**KIR2DL1**	19	95	T	C	32	L	P	-	DOID:1324 / lung cancer	4780	MST/ MaLR	S
**KIR2DL1**	19	328	C	T	110	Q	X	-	DOID:1324 / lung cancer	4780	MST/ MaLR	S
**KIR2DL1**	19	55	G	T	19	A	S	-	DOID:1324 / lung cancer	4780	MST/ MaLR	S
**KIR2DL1**	19	487	G	T	163	E	X	-	DOID:1324 / lung cancer	4780	MST/ MaLR	S
**KIR2DL1**	19	506	G	A	169	R	H	-	DOID:1324 / lung cancer	4780	MST/ MaLR	S
**KIR2DL1**	19	308	C	A	103	S	Y	Gain| Phosphorylation	DOID:1324 / lung cancer	4780	MST/ MaLR	S
**KIR2DL1**	19	310	G	C	104	V	L	Gain| Phosphorylation	DOID:263 / kidney cancer	4780	MST/ MaLR	S
**KIR2DL1**	19	487	G	A	163	E	K	-	DOID:263 / kidney cancer	4780	MST/ MaLR	S
**KIR2DL1**	19	608	A	G	203	H	R	Gain| Phosphorylation	DOID:1324 / lung cancer	4780	MST/ MaLR	S
**KIR2DL1**	19	670	C	A	224	P	T	Gain| Glycosylation	DOID:1324 / lung cancer	4780	MST/ MaLR	S
**KIR2DL1**	19	680	G	T	227	S	I	Gain| Glycosylation	DOID:1324 / lung cancer	4780	MST/ MaLR	S
**ZNF99**	19	1858	C	G	620	P	A	-	DOID:263 / kidney cancer	4673	HERV-W/ LTR17/ HERV17	GE
**ZNF99**	19	2272	G	C	758	E	Q	-	DOID:263 / kidney cancer	4673	HERV-W/ LTR17/ HERV17	GE
**ZNF99**	19	2338	A	G	780	K	E	-	DOID:263 / kidney cancer	4673	HERV-W/ LTR17/ HERV17	GE
**ZNF99**	19	1557	G	C	519	K	N	-	DOID:263 / kidney cancer	4673	HERV-W/ LTR17/ HERV17	GE
**ZNF99**	19	1630	A	T	544	K	X	-	DOID:263 / kidney cancer	4673	HERV-W/ LTR17/ HERV17	GE
**ZNF99**	19	2270	C	G	757	A	G	-	DOID:263 / kidney cancer	4673	HERV-W/ LTR17/ HERV17	GE
**ZNF99**	19	1582	A	C	528	K	Q	-	DOID:263 / kidney cancer	4673	HERV-W/ LTR17/ HERV17	GE

^a^ Position of nucleotide

^b^ Reference of nucleotide

^c^ Variant of nucleotide

^d^ Position of amino acid

^e^ Reference of amino acid

^f^ Variant of nucleotide

^g^ Gamma-retrovirus/Epsilon-retrovirus-related

^h^ Spumavirus-related

#### Survival analysis on 22 key nsSNV in cancer patients

**Fig 5:**
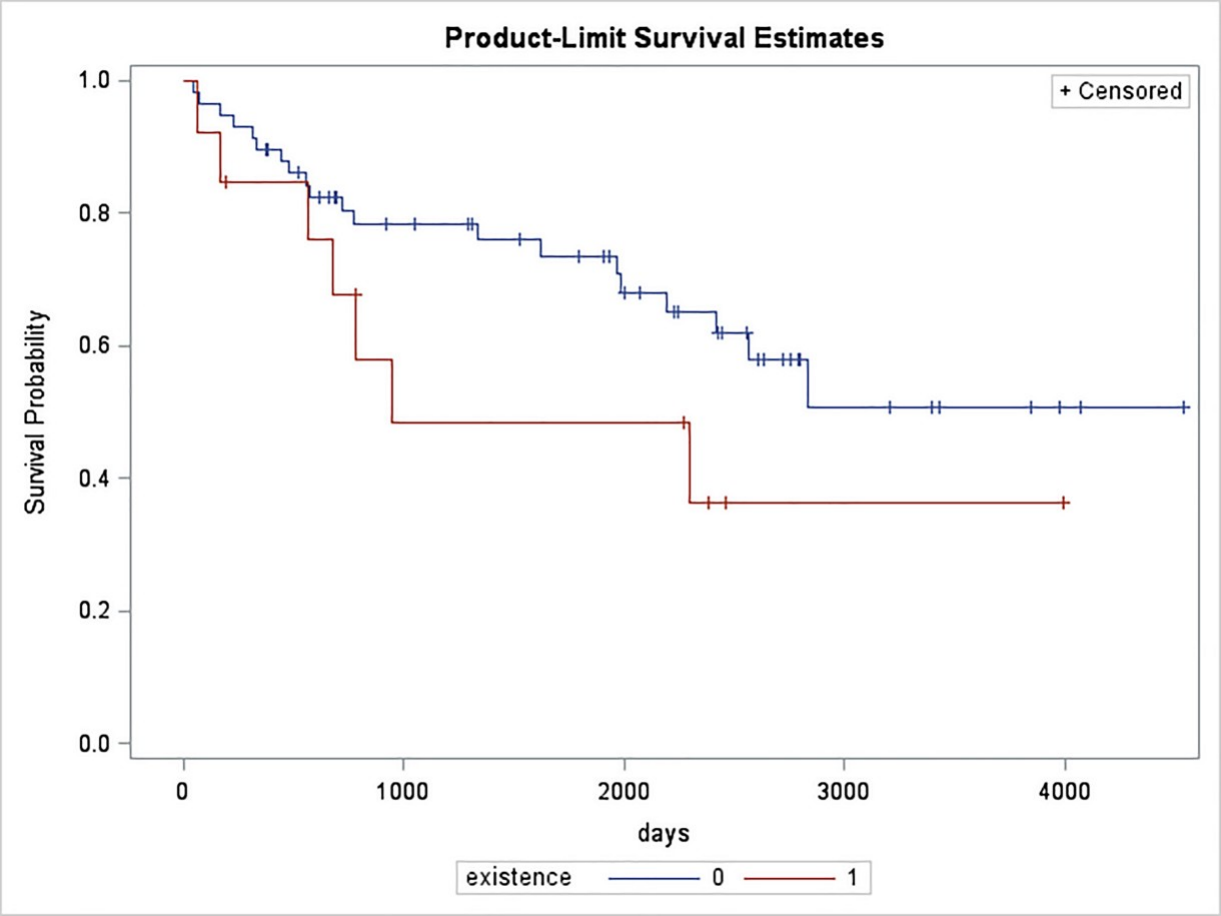
The survival rate in the kidney cancer patients with or without the nsSNV at genomic position 2270 (C to G modification) of ZNF99. Kaplan-Meier plot of kidney cancer patient survival based on the existence of ZNF99 (C2270G). X-axis indicate days of survival. Y-axis indicates survival probability. Red and blue lines indicate survival time of kidney cancer patients with and without such mutation respectively. Log-rank test shows that, comparing with patients with ZNF99(C2270G), patients without this nsSNV survive longer with adjusted p-value of 0.05. The hazard ratio is 2.642.

The total number of nsSNVs in genes which showed significant differential expression in cancer tissues compared to non-tumor tissues were 22 (Table 3). After extracting metadata and clinical data for patients for these 22 nsSNVs, we found one SNV in *ZNF99* to be associated closely with patient survival rate, as shown in Fig 5.

As shown in Fig 5, patients with this nsSNV in *ZNF99* at amino acid position 757 (A to G modification) have a lower survival rate than the patients without this variation. This key amino acid located in position 757 of ZNF99, which triggers abnormal significant under-expression of *ZNF99* in kidney cancer. Additionally, the kidney cancer patients with this key mutation in *ZNF99* have a significant decrease in survival rate (Hazard ratio = 2.642; p = 0.05). We believe this nsSNV could be involved in the progression of cancer and further analysis is warranted to validate this mutation.

### Distribution of SNVs in HERVs from human protein non-coding regions that overlap with DNA functional elements

**Fig 6:**
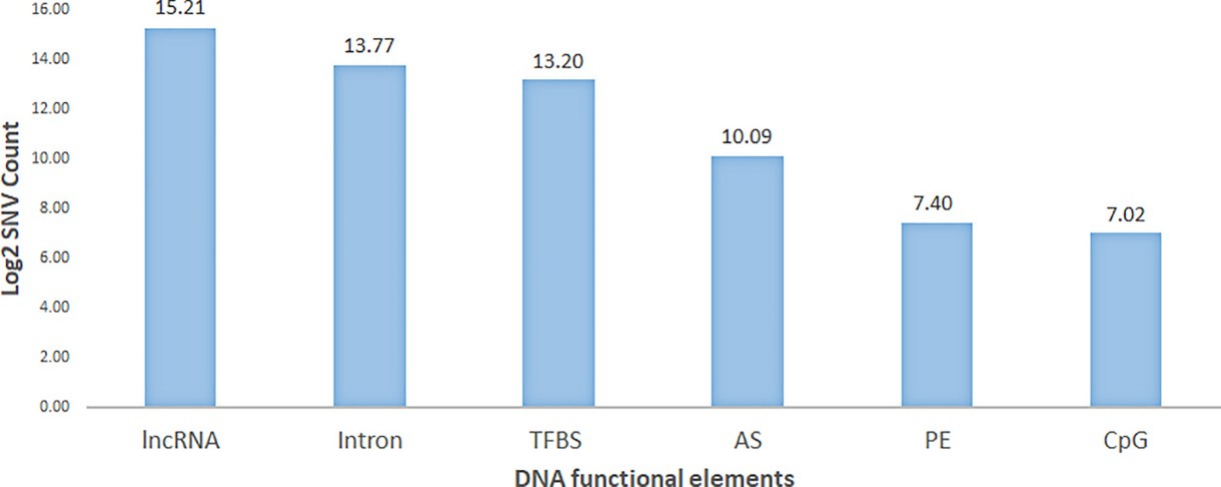
HERV elements on human non-coding region with over-represented SNVs distributed to DNA functional elements. X-axis is different DNA functional elements. Y-axis is Log2Counts (Counts mean the number of SNVs). The proportion of SNVs on DNA functional elements are LncRNA (long non-coding RNA) (60%), Intron (22.2%), TFBS (transcription factor binding site) (15%), AS (alternative splice site) (1.7%), PE (pseudo exon) (0.27%), and CpG (CpG Island) (0.2%).

To investigate the potential functional roles of the HERV elements from protein non-coding regions, with over-represented mutations, we mapped them to our DNA functional elements dataset. We found that 62,575 variants occur in both HERV elements and in at least one DNA functional element (Fig 6). In our results (Fig 6 and S12 Table), the proportion of SNVs within lncRNA, intron, and TFBS is over 97% of all functionally affected SNVs. The top functional element is long non-coding RNA (lncRNA) which contains 60% of these variants. Additionally, other highly represented functional elements are intron (22.2%), transcription factor binding site (TFBS) (15%), alternative splice site (AS) (1.7%), pseudo exon (PE) (0.27%), and CpG Island (0.2%).

**Fig 7:**
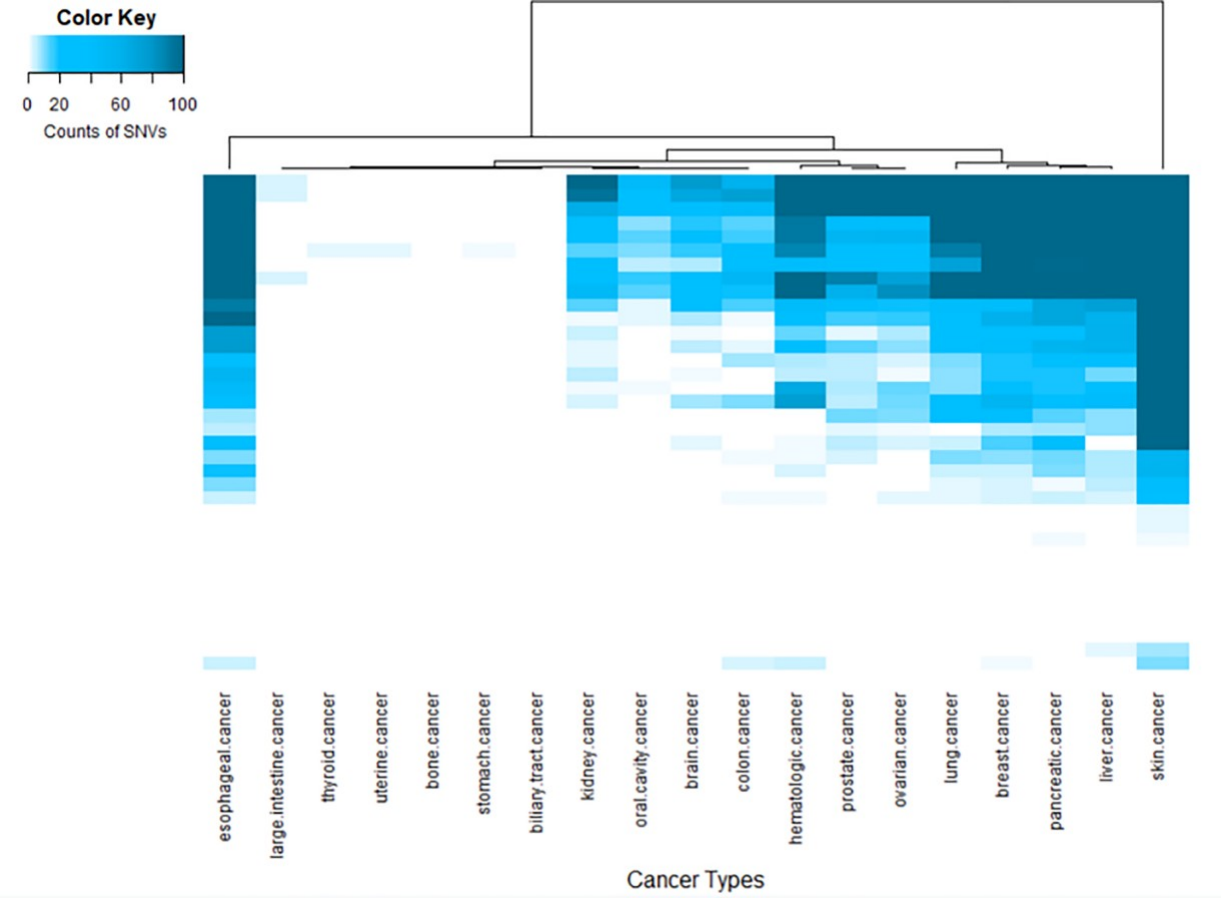
The correlation between HERV-involved DNA functional elements and cancers. Y-axis indicates the SNVs within each HERV class located on each DNA functional elements from top to bottom: SNVs located on HERV elements and DNA functional elements which have combinations containing lncRNA-Can-CI, lncRNA-Can-CII, lncRNA-Can-CIII, lncRNA-Non-CI, lncRNA-Non-CII lncRNA-Non-CIII, Intron-Can-CI, Intron-Can-CII, Intron-Can-CIII, Intron-Non-CI, Intron-Non-CI, Intron-Non-CI, TFBS-Can-CI, TFBS-Can-CII, TFBS-Can-CIII, TFBS-Non-CI, TFBS-Non-CII, TFBS-Non-CIII, AS-Can-CI, AS-Can-CII, AS-Can-CIII, AS-Non-CI, AS-Non-CII, AS-Non-CIII, PE-Can-CI, PE-Can-CII, PE-Can-CIII, PE-Non-CI, PE-Non-CII, PE-Non-CIII, CpG-Can-CI, CpG-Can-CII, CpG-Can-CIII, CpG-Non-CI, CpG-Non-CII, and CpG-Non-CIII. (Can indicates canonical HERV; Non indicates non-conical HERV; Cl indicates Gamma-retrovirus/Epsilon-retrovirus-related HERV; ClI indicates Alpha-retrovirus/ Beta-retrovirus-related HERV; ClII indicates Spumavirus-related HERV; LncRNA indicates long non-coding RNA; TFBS indicates transcription factor binding site; AS indicates alternative splice site; PE indicates pseudo exon; CpG indicates CpG Island) HERV-involved lncRNA, intron, and TFBS are found in skin cancer, esophageal cancer, and liver cancer in either canonical HERVs and non-canonical HERVs.

Cancer and HERV associations were further explored in lncRNA, intron, and TFBS. Fig 7 shows that, HERV mutations in lncRNAs are found in multiple cancers, especially, skin and esophageal cancer. Additionally, HERV mutation hotspots in introns are from at least 10 cancer types. HERV mutations in TFBS is primarily found in skin cancer and breast cancer; especially, the non-canonical Gamma-retrovirus/Epsilon-retrovirus-related (GE) HERV in the TFBS. This study provides a direction to narrow down the HERVs in well-defined DNA functional elements which potentially could play a role in cancer.

## Discussion

In this study, we provide a large-scale and systematic analysis of somatic SNVs in different HERV classes and their subgroups. In our study, we identified four HERV elements with mutation hotspots that overlap with exons of four genes. Those genes are *TNN*, *KIR2DL1*, *ZNF99*, and *OR4K15*. It is well known that LTRs have played a significant role in human gene evolution [76]. Yu et al. have suggested that the mutation hotspot located on the 3’-LTR of HERV-W element may have a regulatory role and might be involved in the activation of neighboring genes and its abnormal expression [30]. A few studies support the involvement of *TNN* and *KIR2DL1* in tumorigenesis. *TNN* is an extracellular matrix protein. It regulates *TGF-beta* to drive breast carcinogenesis and facilitates the migration of cancer cells into bone [77]. KIR2DL1+-HLA-C2+ (human leukocyte antigen) genotype was found in oral cancer patients [78]. In Fig 2, multiple exons (Exon 1–5) of KIR2DL1, which overlap with MaLR/MST element seems associated with oral cancer. A study indicated that the *ZNF99* gene is involved in the transcription of HERV-W/HERV17/LTR17, which has been implicated in the pathogenesis of multiple sclerosis [79]. Interestingly, the patients with multiple sclerosis have an increased risk of lung, liver, hematologic, and bladder cancer [80]. In Fig 2, *ZNF99* corresponding to HERV-W/HERV17/LTR17 are found in few cancers; especially, lung cancer. Importantly, patients with the alteration of amino acid position 757 (A to G modification) in *ZNF99* have a lower survival rate than the patients without this variation as shown in Fig 5. Overall, our data indicated over-representative SNVs (hotspots) strengthen the relationship between these four possible HERV-involved genes and multiple cancer types.

We found the SNVs within the HERV-H/LTR7 element are the major family in Gamma-retrovirus/Epsilon-retrovirus-related (GE) retroviruses—both canonical and non-canonical HERVs (Fig 3). Liang et al [75] indicates that non-*env*-related transcripts of HERV-H are up-regulated in colon cancer cell lines because of abnormal methylation. A few SNVs in HERV-H/LTR7—both canonical and non-canonical—are associated with colon cancer and provides evidence that HERV-H/LTR7 could be involved in tumorigenesis. Additionally, the HML-2/HERV-K/LTR5 element, which is of Alpha-retrovirus/Beta-retrovirus-related (AB) retroviruses, appears in the blood of patients with breast and lymphoma cancer [81]. Based on the results and relevant research, HERV elements on the non-coding region may play a crucial role in cancer.

SNVs in regulatory DNA elements could broadly affect transcription by altering enhancer and promoter activity or chromatin states, leading to abnormal expression in diseases [82]. Further research supports DNA functional elements such as lncRNAs could be involved in carcinogenesis because mutations are located in the DNA functional region [83, 84]. Several SNVs are implicated in the expression of cancer-associated lncRNAs—including *CCAT2* in colorectal cancers [85] and *PCAT-1* in prostate cancer [86]. Additionally, gamma-like retrovirus, one virus of Gamma-retrovirus/Epsilon-retrovirus-related (GE) HERVs, has a connection to carcinogenesis through lncRNA [87]. HERVs that map to lncRNAs which contain multiple somatic SNVs could play a potential role in carcinogenesis, especially, skin and esophageal cancer. Similarly HERVs mapped to introns have been implicated with cancer formation [88]. This was the first report that an intronic mutation was related to the development of cancer [88]. Moreover, copy number variants of the TFBS which is involved in a proliferation effector-gene and an apoptosis effector-gene are highly associated with melanoma and breast cancer [88].

One of the limitations is the lack of comprehensive SNV datasets for protein non-coding regions. Due to the cost of human whole genome sequencing (WGS) in cancer patients, majority of the data is from whole exome sequencing (WEX) instead of WGS. In order to overcome inherent limitations in the data, more functional analysis is required for supporting the potential mechanism of HERV elements’ association with cancers. Another limitation is the mutation calling within repetitive region. To effectively minimize issues that could potentially cause bias in the sequencing result, the review offers insightful perspectives and potential solutions in the alignment step [49]. Firstly, the pipeline of TCGA and other resources that we used adopt similar mate-pair information from reads that were sequenced in pairs for alignment steps to account for repetitive regions in human genome [50]. Duplicate reads are also marked after alignment, sorting and merging via Picard Tools MarkDuplicate function. This is based on the similarity of the 5' and 3' ends of the strands. This is used to protect against a single region being sequenced in much higher quantity than the genome at large; but also help mitigate repetitive regions by essentially labeling them as duplicates and then removing them from consideration. This general same principle also applies to repetitive regions to decrease the chance that all the reads get mapped to one region and variants are called off of that even though the reads are from different very similar regions. Another strategy for handling repeats “is to compute statistics on the depth of coverage for each contig” quoted from Treangen’s paper [49]. The TCGA pipeline checks for higher than 50X sequence coverage for mutation calling (https://docs.gdc.cancer.gov/Data/Introduction/). TCGA and ICGC data has been used to study regulatory elements including untranslated regions, splice sites and non-coding RNA with repeats [51]. We believe that although there is a possibility that the repeats might result in misalignments, the methods used to determine these SNVs are robust enough to provide reliable mutation calls and calculate SNV hotspots.

This study has identified mutational hotspots in HERVs and attempts to rank HERVs which might be associated with cancer. Although, survival analysis is performed with one mutation, it is clear that there can be other mutations in HERVs which can have a profound impact on cancer progression. The ultimate goal of this study is to provide directions and suggestions for further research related to deciphering the role of HERVs in cancer.

## Conclusion

In this study, we explored the correlation between HERV elements on human protein coding and non-coding regions and multiple cancers based on SNV hotspots. In the HERV element on human protein coding regions, we found four HERV elements that had over-represented nsSNVs and also overlapped with exons of four genes. Additionally, these four HERV elements were associated with at least 14 cancer types—notably skin and lung cancer. We showed that kidney cancer patients with the specific amino acid mutation A757G in ZNF99 within the HERV-W/LTR17/HERV17 element had a lower survival rate based on a survival analysis. We believe that this key mutation could play an important role in kidney cancer.

In the HERV element on human protein non-coding regions, we found 357 canonical and 431 non-canonical HERV elements across different classes which had significantly elevated SNVs counts. All SNVs within these 788 HERV elements overlapped with six DNA functional element groups. HERVs involved in the functional groups lncRNA, introns, and TFBS were shown to be associated with skin, esophageal, and liver cancer. Since we were able to narrow the number of cancer-related SNVs within HERV elements into six groups, we believe these are high-priority experimental targets for studying the molecular mechanisms in cancer progression.

## Supporting information

S1 File a~x The distribution of nsSNVs in protein coding region and SNVs in non-coding region by chromosome 1–22, X, Y. X-axis indicates the genomic positions of chromosome. Y-axis indicates the count of mutations. This Manhattan plot represents the distribution of mutations in all positions of the chromosome except for the centromere (empty region).
(DOCX)

S1 Fig Each HERV element with count of somatic SNVs within non-coding region. X-axis indicates cancer types. Y-axis indicates Log2Counts (counts mean the number of SNVs). Box plot represents the number of SNVs located in the HERV elements.
(TIFF)

S1 Table HERV elements dataset. (XLSX)

S2 Table List of 39 canonical and 31 non-canonical HERV clades found in GRCh37/hg19. (XLSX)

S3 Table The number of SNVs in every 1000 bases and their observed ratio in 22+XY chromosomes. (XLSX)

S4 Table HERV elements on human protein coding region with significant representation of nsSNVs. (XLSX)

S5 Table HERV elements on human non-coding region with significant representation of SNVs. (XLSX)

S6 Table Somatic nsSNVs in HERV elements on human protein coding region. (XLSX)

S7 Table Somatic SNVs in HERV elements on human non-coding region. (XLSX)

S8 Table The regions of exon in four genes overlap to HERV elements. (XLSX)

S9 Table 492 nsSNVs in HERV elements on human protein coding region with significant over-representation. (XLSX)

S10 Table 193,439 SNVs in HERV elements on human non-coding region with significant over-representation. (XLSX)

S11 Table The total count of nsSNVs in functional sites within HERV elements. (XLSX)

S12 Table SNVs in HERV elements on human non-coding region overlap to DNA functional elements. (XLSX)

## Abbreviations

HERV: human endogenous retrovirus
LTRs: long terminal repeats
nsSNV: non-synonymous single-nucleotide variation
SNV: single-nucleotide variation
TNN: Tenascin-N
KIR2DL1: Killer Cell Immunoglobulin Like Receptor, Two Ig Domains Long Cytoplasmic Tail 1
OR4K15: Olfactory Receptor Family 4 Subfamily K Member 15
ZNF99: Zinc Finger Protein 99
PTM: post translational modification
lncRNA: long non-coding RNA
TFBS: transcriptional factor binding site
AS: alternative splice site
PE: pseudo exon

## References

[1] Bannert N, Kurth R. The evolutionary dynamics of human endogenous retroviral families. Annual review of genomics and human genetics. 2006;7:149–73. 10.1146/annurev.genom.7.080505.115700 . 16722807

[2] Katzourakis A, Rambaut A, Pybus OG. The evolutionary dynamics of endogenous retroviruses. Trends in microbiology. 2005;13(10):463–8. 10.1016/j.tim.2005.08.004 . 16109487

[3] Goff SP. Host factors exploited by retroviruses. Nature reviews Microbiology. 2007;5(4):253–63. 10.1038/nrmicro1541 . 17325726

[4] Benachenhou F, Blikstad V, Blomberg J. The phylogeny of orthoretroviral long terminal repeats (LTRs). Gene. 2009;448(2):134–8. 10.1016/j.gene.2009.07.002 . 19595747

[5] Stoye JP. Studies of endogenous retroviruses reveal a continuing evolutionary saga. Nature reviews Microbiology. 2012;10(6):395–406. 10.1038/nrmicro2783 . 22565131

[6] Lander ES, Linton LM, Birren B, Nusbaum C, Zody MC, Baldwin J, et al Initial sequencing and analysis of the human genome. Nature. 2001;409(6822):860–921. 10.1038/35057062 . 11237011

[7] Wilkinson DA MD, Leong JC. Endogenous human retroviruses. J L, editor. New York: Plenum Press; 1994.

[8] Tristem M. Identification and characterization of novel human endogenous retrovirus families by phylogenetic screening of the human genome mapping project database. J Virol. 2000;74(8):3715–30. 10729147 10.1128/jvi.74.8.3715-3730.2000 PMC111881

[9] Gifford RJ, Blomberg J, Coffin JM, Fan H, Heidmann T, Mayer J, et al Nomenclature for endogenous retrovirus (ERV) loci. Retrovirology. 2018;15(1):59 10.1186/s12977-018-0442-1 30153831 PMC6114882

[10] Andersson ML, Lindeskog M, Medstrand P, Westley B, May F, Blomberg J. Diversity of human endogenous retrovirus class II-like sequences. The Journal of general virology. 1999;80 (Pt 1):255–60. 10.1099/0022-1317-80-1-255 . 9934709

[11] Blomberg J, Benachenhou F, Blikstad V, Sperber G, Mayer J. Classification and nomenclature of endogenous retroviral sequences (ERVs): problems and recommendations. Gene. 2009;448(2):115–23. 10.1016/j.gene.2009.06.007 . 19540319

[12] Mayer J, Blomberg J, Seal RL. A revised nomenclature for transcribed human endogenous retroviral loci. Mobile DNA. 2011;2(1):7 10.1186/1759-8753-2-7 21542922 PMC3113919

[13] Vargiu L, Rodriguez-Tome P, Sperber GO, Cadeddu M, Grandi N, Blikstad V, et al Classification and characterization of human endogenous retroviruses; mosaic forms are common. Retrovirology. 2016;13:7 10.1186/s12977-015-0232-y 26800882 PMC4724089

[14] Jurka J. Repbase update: a database and an electronic journal of repetitive elements. Trends in genetics: TIG. 2000;16(9):418–20. . 10973072 10.1016/s0168-9525(00)02093-x

[15] Jurka J, Kapitonov VV, Pavlicek A, Klonowski P, Kohany O, Walichiewicz J. Repbase Update, a database of eukaryotic repetitive elements. Cytogenetic and genome research. 2005;110(1–4):462–7. 10.1159/000084979 . 16093699

[16] Sperber GO, Airola T, Jern P, Blomberg J. Automated recognition of retroviral sequences in genomic data—RetroTector. Nucleic Acids Res. 2007;35(15):4964–76. 10.1093/nar/gkm515 17636050 PMC1976444

[17] Elfaitouri A, Shao X, Mattsson Ulfstedt J, Muradrasoli S, Bolin Wiener A, Golbob S, et al Murine gammaretrovirus group G3 was not found in Swedish patients with myalgic encephalomyelitis/chronic fatigue syndrome and fibromyalgia. PloS one. 2011;6(10):e24602 10.1371/journal.pone.0024602 22022360 PMC3192035

[18] Jern P, Sperber GO, Blomberg J. Use of endogenous retroviral sequences (ERVs) and structural markers for retroviral phylogenetic inference and taxonomy. Retrovirology. 2005;2:50 10.1186/1742-4690-2-50 16092962 PMC1224870

[19] de Parseval N, Lazar V, Casella JF, Benit L, Heidmann T. Survey of human genes of retroviral origin: identification and transcriptome of the genes with coding capacity for complete envelope proteins. Journal of virology. 2003;77(19):10414–22. 10.1128/JVI.77.19.10414-10422.2003 12970426 PMC228468

[20] Grow EJ, Flynn RA, Chavez SL, Bayless NL, Wossidlo M, Wesche DJ, et al Intrinsic retroviral reactivation in human preimplantation embryos and pluripotent cells. Nature. 2015;522(7555):221–5. 10.1038/nature14308 25896322 PMC4503379

[21] Perron H, Lazarini F, Ruprecht K, Pechoux-Longin C, Seilhean D, Sazdovitch V, et al Human endogenous retrovirus (HERV)-W ENV and GAG proteins: physiological expression in human brain and pathophysiological modulation in multiple sclerosis lesions. Journal of neurovirology. 2005;11(1):23–33. 10.1080/13550280590901741 . 15804956

[22] Galli UM, Sauter M, Lecher B, Maurer S, Herbst H, Roemer K, et al Human endogenous retrovirus rec interferes with germ cell development in mice and may cause carcinoma in situ, the predecessor lesion of germ cell tumors. Oncogene. 2005;24(19):3223–8. 10.1038/sj.onc.1208543 . 15735668

[23] Strick R, Ackermann S, Langbein M, Swiatek J, Schubert SW, Hashemolhosseini S, et al Proliferation and cell-cell fusion of endometrial carcinoma are induced by the human endogenous retroviral Syncytin-1 and regulated by TGF-beta. Journal of molecular medicine. 2007;85(1):23–38. 10.1007/s00109-006-0104-y . 17066266

[24] Stauffer Y, Theiler G, Sperisen P, Lebedev Y, Jongeneel CV. Digital expression profiles of human endogenous retroviral families in normal and cancerous tissues. Cancer immunity. 2004;4:2 . 14871062

[25] Balestrieri E, Arpino C, Matteucci C, Sorrentino R, Pica F, Alessandrelli R, et al HERVs expression in Autism Spectrum Disorders. PloS one. 2012;7(11):e48831 10.1371/journal.pone.0048831 23155411 PMC3498248

[26] Armbruester V, Sauter M, Roemer K, Best B, Hahn S, Nty A, et al Np9 protein of human endogenous retrovirus K interacts with ligand of numb protein X. Journal of virology. 2004;78(19):10310–9. 10.1128/JVI.78.19.10310-10319.2004 15367597 PMC516385

[27] Denne M, Sauter M, Armbruester V, Licht JD, Roemer K, Mueller-Lantzsch N. Physical and functional interactions of human endogenous retrovirus proteins Np9 and rec with the promyelocytic leukemia zinc finger protein. Journal of virology. 2007;81(11):5607–16. 10.1128/JVI.02771-06 17360752 PMC1900259

[28] Sin HS, Huh JW, Kim DS, Kang DW, Min DS, Kim TH, et al Transcriptional control of the HERV-H LTR element of the GSDML gene in human tissues and cancer cells. Archives of virology. 2006;151(10):1985–94. 10.1007/s00705-006-0764-5 . 16625320

[29] Romanish MT, Lock WM, van de Lagemaat LN, Dunn CA, Mager DL. Repeated recruitment of LTR retrotransposons as promoters by the anti-apoptotic locus NAIP during mammalian evolution. PLoS genetics. 2007;3(1):e10 10.1371/journal.pgen.0030010 17222062 PMC1781489

[30] Yu H, Liu T, Zhao Z, Chen Y, Zeng J, Liu S, et al Mutations in 3'-long terminal repeat of HERV-W family in chromosome 7 upregulate syncytin-1 expression in urothelial cell carcinoma of the bladder through interacting with c-Myb. Oncogene. 2014;33(30):3947–58. 10.1038/onc.2013.366 . 24013223

[31] Glinsky GV. Single cell genomics reveals activation signatures of endogenous SCAR's networks in aneuploid human embryos and clinically intractable malignant tumors. Cancer letters. 2016;381(1):176–93. 10.1016/j.canlet.2016.08.001 . 27497790

[32] Kuntzer J, Eggle D, Klostermann S, Burtscher H. Human variation databases. Database: the journal of biological databases and curation. 2010;2010:baq015 10.1093/database/baq015 20639550 PMC2911800

[33] Genomes Project C, Abecasis GR, Auton A, Brooks LD, DePristo MA, Durbin RM, et al An integrated map of genetic variation from 1,092 human genomes. Nature. 2012;491(7422):56–65. 10.1038/nature11632 23128226 PMC3498066

[34] Lehrach H. DNA sequencing methods in human genetics and disease research. F1000prime reports. 2013;5:34 10.12703/P5-34 24049638 PMC3768324

[35] Wu TJ, Shamsaddini A, Pan Y, Smith K, Crichton DJ, Simonyan V, et al A framework for organizing cancer-related variations from existing databases, publications and NGS data using a High-performance Integrated Virtual Environment (HIVE). Database (Oxford). 2014;2014:bau022 10.1093/database/bau022 24667251 PMC3965850

[36] Kandoth C, McLellan MD, Vandin F, Ye K, Niu B, Lu C, et al Mutational landscape and significance across 12 major cancer types. Nature. 2013;502(7471):333–9. 10.1038/nature12634 24132290 PMC3927368

[37] Reimand J, Wagih O, Bader GD. The mutational landscape of phosphorylation signaling in cancer. Sci Rep. 2013;3:2651 10.1038/srep02651 24089029 PMC3788619

[38] Miller ML, Reznik E, Gauthier NP, Aksoy BA, Korkut A, Gao J, et al Pan-Cancer Analysis of Mutation Hotspots in Protein Domains. Cell Syst. 2015;1(3):197–209. 10.1016/j.cels.2015.08.014 27135912 PMC4982675

[39] Dingerdissen HM, Torcivia-Rodriguez J, Hu Y, Chang TC, Mazumder R, Kahsay R. BioMuta and BioXpress: mutation and expression knowledgebases for cancer biomarker discovery. Nucleic acids research. 2018;46(D1):D1128–D36. 10.1093/nar/gkx907 30053270 PMC5753215

[40] Cancer Genome Atlas Research N, Weinstein JN, Collisson EA, Mills GB, Shaw KR, Ozenberger BA, et al The Cancer Genome Atlas Pan-Cancer analysis project. Nature genetics. 2013;45(10):1113–20. 10.1038/ng.2764 24071849 PMC3919969

[41] Landrum MJ, Lee JM, Riley GR, Jang W, Rubinstein WS, Church DM, et al ClinVar: public archive of relationships among sequence variation and human phenotype. Nucleic acids research. 2014;42(Database issue):D980–5. 10.1093/nar/gkt1113 24234437 PMC3965032

[42] Forbes SA, Beare D, Gunasekaran P, Leung K, Bindal N, Boutselakis H, et al COSMIC: exploring the world's knowledge of somatic mutations in human cancer. Nucleic acids research. 2015;43(Database issue):D805–11. 10.1093/nar/gku1075 25355519 PMC4383913

[43] International Cancer Genome C, Hudson TJ, Anderson W, Artez A, Barker AD, Bell C, et al International network of cancer genome projects. Nature. 2010;464(7291):993–8. 10.1038/nature08987 20393554 PMC2902243

[44] Gonzalez-Perez A, Perez-Llamas C, Deu-Pons J, Tamborero D, Schroeder MP, Jene-Sanz A, et al IntOGen-mutations identifies cancer drivers across tumor types. Nature methods. 2013;10(11):1081–2. 10.1038/nmeth.2642 . 24037244 PMC5758042

[45] Boutet E, Lieberherr D, Tognolli M, Schneider M, Bansal P, Bridge AJ, et al UniProtKB/Swiss-Prot, the Manually Annotated Section of the UniProt KnowledgeBase: How to Use the Entry View. Methods in molecular biology. 2016;1374:23–54. 10.1007/978-1-4939-3167-5_2 . 26519399

[46] Cibulskis K, Lawrence MS, Carter SL, Sivachenko A, Jaffe D, Sougnez C, et al Sensitive detection of somatic point mutations in impure and heterogeneous cancer samples. Nature biotechnology. 2013;31(3):213–9. 10.1038/nbt.2514 23396013 PMC3833702

[47] Saunders CT, Wong WS, Swamy S, Becq J, Murray LJ, Cheetham RK. Strelka: accurate somatic small-variant calling from sequenced tumor-normal sample pairs. Bioinformatics. 2012;28(14):1811–7. 10.1093/bioinformatics/bts271 . 22581179

[48] Alioto TS, Buchhalter I, Derdak S, Hutter B, Eldridge MD, Hovig E, et al A comprehensive assessment of somatic mutation detection in cancer using whole-genome sequencing. Nature communications. 2015;6:10001 10.1038/ncomms10001 26647970 PMC4682041

[49] Treangen TJ, Salzberg SL. Repetitive DNA and next-generation sequencing: computational challenges and solutions. Nature reviews Genetics. 2011;13(1):36–46. 10.1038/nrg3117 22124482 PMC3324860

[50] Li H, Durbin R. Fast and accurate short read alignment with Burrows-Wheeler transform. Bioinformatics. 2009;25(14):1754–60. 10.1093/bioinformatics/btp324 19451168 PMC2705234

[51] Diederichs S, Bartsch L, Berkmann JC, Frose K, Heitmann J, Hoppe C, et al The dark matter of the cancer genome: aberrations in regulatory elements, untranslated regions, splice sites, non-coding RNA and synonymous mutations. EMBO molecular medicine. 2016;8(5):442–57. 10.15252/emmm.201506055 26992833 PMC5126213

[52] Chan PP, Lowe TM. GtRNAdb: a database of transfer RNA genes detected in genomic sequence. Nucleic acids research. 2009;37(Database issue):D93–7. 10.1093/nar/gkn787 18984615 PMC2686519

[53] Eddy SR, Durbin R. RNA sequence analysis using covariance models. Nucleic acids research. 1994;22(11):2079–88. 8029015 10.1093/nar/22.11.2079 PMC308124

[54] Lowe TM, Eddy SR. tRNAscan-SE: a program for improved detection of transfer RNA genes in genomic sequence. Nucleic acids research. 1997;25(5):955–64. 9023104 10.1093/nar/25.5.955 PMC146525

[55] Griffith OL, Montgomery SB, Bernier B, Chu B, Kasaian K, Aerts S, et al ORegAnno: an open-access community-driven resource for regulatory annotation. Nucleic acids research. 2008;36(Database issue):D107–13. 10.1093/nar/gkm967 18006570 PMC2239002

[56] Montgomery SB, Griffith OL, Sleumer MC, Bergman CM, Bilenky M, Pleasance ED, et al ORegAnno: an open access database and curation system for literature-derived promoters, transcription factor binding sites and regulatory variation. Bioinformatics. 2006;22(5):637–40. 10.1093/bioinformatics/btk027 . 16397004

[57] Griffiths-Jones S, Grocock RJ, van Dongen S, Bateman A, Enright AJ. miRBase: microRNA sequences, targets and gene nomenclature. Nucleic acids research. 2006;34(Database issue):D140–4. 10.1093/nar/gkj112 16381832 PMC1347474

[58] Zhang Z, Harrison PM, Liu Y, Gerstein M. Millions of years of evolution preserved: a comprehensive catalog of the processed pseudogenes in the human genome. Genome research. 2003;13(12):2541–58. 10.1101/gr.1429003 14656962 PMC403796

[59] Zheng D, Zhang Z, Harrison PM, Karro J, Carriero N, Gerstein M. Integrated pseudogene annotation for human chromosome 22: evidence for transcription. Journal of molecular biology. 2005;349(1):27–45. 10.1016/j.jmb.2005.02.072 . 15876366

[60] Pennacchio LA, Ahituv N, Moses AM, Prabhakar S, Nobrega MA, Shoukry M, et al In vivo enhancer analysis of human conserved non-coding sequences. Nature. 2006;444(7118):499–502. 10.1038/nature05295 . 17086198

[61] Lestrade L, Weber MJ. snoRNA-LBME-db, a comprehensive database of human H/ACA and C/D box snoRNAs. Nucleic acids research. 2006;34(Database issue):D158–62. 10.1093/nar/gkj002 16381836 PMC1347365

[62] Gerstein MB, Kundaje A, Hariharan M, Landt SG, Yan KK, Cheng C, et al Architecture of the human regulatory network derived from ENCODE data. Nature. 2012;489(7414):91–100. 10.1038/nature11245 22955619 PMC4154057

[63] Wang J, Zhuang J, Iyer S, Lin XY, Greven MC, Kim BH, et al Factorbook.org: a Wiki-based database for transcription factor-binding data generated by the ENCODE consortium. Nucleic acids research. 2013;41(Database issue):D171–6. 10.1093/nar/gks1221 23203885 PMC3531197

[64] Volders PJ, Verheggen K, Menschaert G, Vandepoele K, Martens L, Vandesompele J, et al An update on LNCipedia: a database for annotated human lncRNA sequences. Nucleic acids research. 2015;43(Database issue):D174–80. 10.1093/nar/gku1060 25378313 PMC4383901

[65] Karagiannis K, Simonyan V, Mazumder R. SNVDis: a proteome-wide analysis service for evaluating nsSNVs in protein functional sites and pathways. Genomics, proteomics & bioinformatics. 2013;11(2):122–6. 10.1016/j.gpb.2012.10.003 23618375 PMC3807806

[66] McElroy K, Zagordi O, Bull R, Luciani F, Beerenwinkel N. Accurate single nucleotide variant detection in viral populations by combining probabilistic clustering with a statistical test of strand bias. BMC genomics. 2013;14:501 10.1186/1471-2164-14-501 23879730 PMC3848937

[67] Mi H, Thomas P. PANTHER pathway: an ontology-based pathway database coupled with data analysis tools. Methods in molecular biology. 2009;563:123–40. 10.1007/978-1-60761-175-2_7 . 19597783 PMC6608593

[68] Pan Y, Karagiannis K, Zhang H, Dingerdissen H, Shamsaddini A, Wan Q, et al Human germline and pan-cancer variomes and their distinct functional profiles. Nucleic acids research. 2014;42(18):11570–88. 10.1093/nar/gku772 25232094 PMC4191387

[69] Gustedt J. Efficient sampling of random permutations. Journal of Discrete Algorithms. 2008;6. Epub 139.

[70] Wan Q, Dingerdissen H, Fan Y, Gulzar N, Pan Y, Wu TJ, et al BioXpress: an integrated RNA-seq-derived gene expression database for pan-cancer analysis. Database: the journal of biological databases and curation. 2015;2015. 10.1093/database/bav019 25819073 PMC4377087

[71] Love MI, Huber W, Anders S. Moderated estimation of fold change and dispersion for RNA-seq data with DESeq2. Genome biology. 2014;15(12):550 10.1186/s13059-014-0550-8 25516281 PMC4302049

[72] Wu TJ, Schriml LM, Chen QR, Colbert M, Crichton DJ, Finney R, et al Generating a focused view of disease ontology cancer terms for pan-cancer data integration and analysis. Database: the journal of biological databases and curation. 2015;2015:bav032 10.1093/database/bav032 25841438 PMC4385274

[73] George B, Seals S, Aban I. Survival analysis and regression models. Journal of nuclear cardiology: official publication of the American Society of Nuclear Cardiology. 2014;21(4):686–94. 10.1007/s12350-014-9908-2 24810431 PMC4111957

[74] Gibb EA, Warren RL, Wilson GW, Brown SD, Robertson GA, Morin GB, et al Activation of an endogenous retrovirus-associated long non-coding RNA in human adenocarcinoma. Genome medicine. 2015;7(1):22 10.1186/s13073-015-0142-6 25821520 PMC4375928

[75] Liang Q, Xu Z, Xu R, Wu L, Zheng S. Expression patterns of non-coding spliced transcripts from human endogenous retrovirus HERV-H elements in colon cancer. PloS one. 2012;7(1):e29950 10.1371/journal.pone.0029950 22238681 PMC3253121

[76] Piriyapongsa J, Polavarapu N, Borodovsky M, McDonald J. Exonization of the LTR transposable elements in human genome. BMC genomics. 2007;8:291 10.1186/1471-2164-8-291 17725822 PMC2008291

[77] Chiovaro F, Martina E, Bottos A, Scherberich A, Hynes NE, Chiquet-Ehrismann R. Transcriptional regulation of tenascin-W by TGF-beta signaling in the bone metastatic niche of breast cancer cells. International journal of cancer. 2015;137(8):1842–54. 10.1002/ijc.29565 25868708 PMC5029769

[78] Dutta A, Saikia N, Phookan J, Baruah MN, Baruah S. Association of killer cell immunoglobulin-like receptor gene 2DL1 and its HLA-C2 ligand with family history of cancer in oral squamous cell carcinoma. Immunogenetics. 2014;66(7–8):439–48. 10.1007/s00251-014-0778-1 . 24818561

[79] Schmitt K, Richter C, Backes C, Meese E, Ruprecht K, Mayer J. Comprehensive analysis of human endogenous retrovirus group HERV-W locus transcription in multiple sclerosis brain lesions by high-throughput amplicon sequencing. Journal of virology. 2013;87(24):13837–52. 10.1128/JVI.02388-13 24109235 PMC3838257

[80] Onishi A, Sugiyama D, Kumagai S, Morinobu A. Cancer incidence in systemic sclerosis: meta-analysis of population-based cohort studies. Arthritis and rheumatism. 2013;65(7):1913–21. 10.1002/art.37969 . 23576072

[81] Contreras-Galindo R, Kaplan MH, Leissner P, Verjat T, Ferlenghi I, Bagnoli F, et al Human endogenous retrovirus K (HML-2) elements in the plasma of people with lymphoma and breast cancer. Journal of virology. 2008;82(19):9329–36. 10.1128/JVI.00646-08 18632860 PMC2546968

[82] Latos PA, Pauler FM, Koerner MV, Senergin HB, Hudson QJ, Stocsits RR, et al Airn transcriptional overlap, but not its lncRNA products, induces imprinted Igf2r silencing. Science. 2012;338(6113):1469–72. 10.1126/science.1228110 . 23239737

[83] Calin GA, Liu CG, Ferracin M, Hyslop T, Spizzo R, Sevignani C, et al Ultraconserved regions encoding ncRNAs are altered in human leukemias and carcinomas. Cancer cell. 2007;12(3):215–29. 10.1016/j.ccr.2007.07.027 . 17785203

[84] Schmitt AM, Chang HY. Long Noncoding RNAs in Cancer Pathways. Cancer cell. 2016;29(4):452–63. 10.1016/j.ccell.2016.03.010 27070700 PMC4831138

[85] Xiang JF, Yin QF, Chen T, Zhang Y, Zhang XO, Wu Z, et al Human colorectal cancer-specific CCAT1-L lncRNA regulates long-range chromatin interactions at the MYC locus. Cell research. 2014;24(5):513–31. 10.1038/cr.2014.35 24662484 PMC4011346

[86] Ling H, Spizzo R, Atlasi Y, Nicoloso M, Shimizu M, Redis RS, et al CCAT2, a novel noncoding RNA mapping to 8q24, underlies metastatic progression and chromosomal instability in colon cancer. Genome research. 2013;23(9):1446–61. 10.1101/gr.152942.112 23796952 PMC3759721

[87] Gilroy KL, Terry A, Naseer A, de Ridder J, Allahyar A, Wang W, et al Gamma-Retrovirus Integration Marks Cell Type-Specific Cancer Genes: A Novel Profiling Tool in Cancer Genomics. PloS one. 2016;11(4):e0154070 10.1371/journal.pone.0154070 27097319 PMC4838236

[88] Sasani F, Baghban F, Nikbakht Brujeni GH, Kazemi M. TP53 intronic mutations in bovine enzootic hematuria-associated urinary bladder tumors. Vet Pathol. 2013;50(3):543–7. 10.1177/0300985812469632 . 23242803

