# Supplementary material for: Investigation of somatic single nucleotide variations in human endogenous retrovirus elements and their potential association with cancer
Source: PLoS One. 2019 Apr 1;14(4):e0213770. doi: 10.1371/journal.pone.0213770 (PMC6443178; doi:10.1371/journal.pone.0213770)

Table S1. The table below shows the ratio between the number of non-coding and coding SNVs in each chromosome.

| Chr | No. of variant in  Coding region | No. of variant in Non-coding region | Ratio (Non-coding region/Coding region) |
| --- | --- | --- | --- |
| 1 | 300772 | 4785255 | 15.9099085 |
| 10 | 118898 | 2575104 | 21.65809349 |
| 11 | 167851 | 2783496 | 16.58313623 |
| 12 | 156440 | 2651239 | 16.94732166 |
| 13 | 54919 | 2010370 | 36.60609261 |
| 14 | 80332 | 1950291 | 24.27788428 |
| 15 | 84919 | 1603806 | 18.88630342 |
| 16 | 99234 | 1804273 | 18.18200415 |
| 17 | 169286 | 1695612 | 10.01625651 |
| 18 | 48160 | 1557871 | 32.34781977 |
| 19 | 171607 | 1645532 | 9.588956161 |
| 2 | 237885 | 4829461 | 20.30166257 |
| 20 | 72772 | 1342812 | 18.45231683 |
| 21 | 30692 | 813512 | 26.50566923 |
| 22 | 48585 | 804668 | 16.56206648 |
| 3 | 173267 | 3974964 | 22.94126406 |
| 4 | 124920 | 3902210 | 31.23767211 |
| 5 | 141261 | 3684644 | 26.08394391 |
| 6 | 153726 | 3350938 | 21.79812133 |
| 7 | 142664 | 3591571 | 25.17503365 |
| 8 | 111568 | 3332698 | 29.8714506 |
| 9 | 104971 | 2193312 | 20.89445656 |
| X | 72527 | 2230458 | 30.75348491 |
| Y | 631 | 91192 | 144.5198098 |

Figure S1-a: Distribution of SNVs in chromosome 1


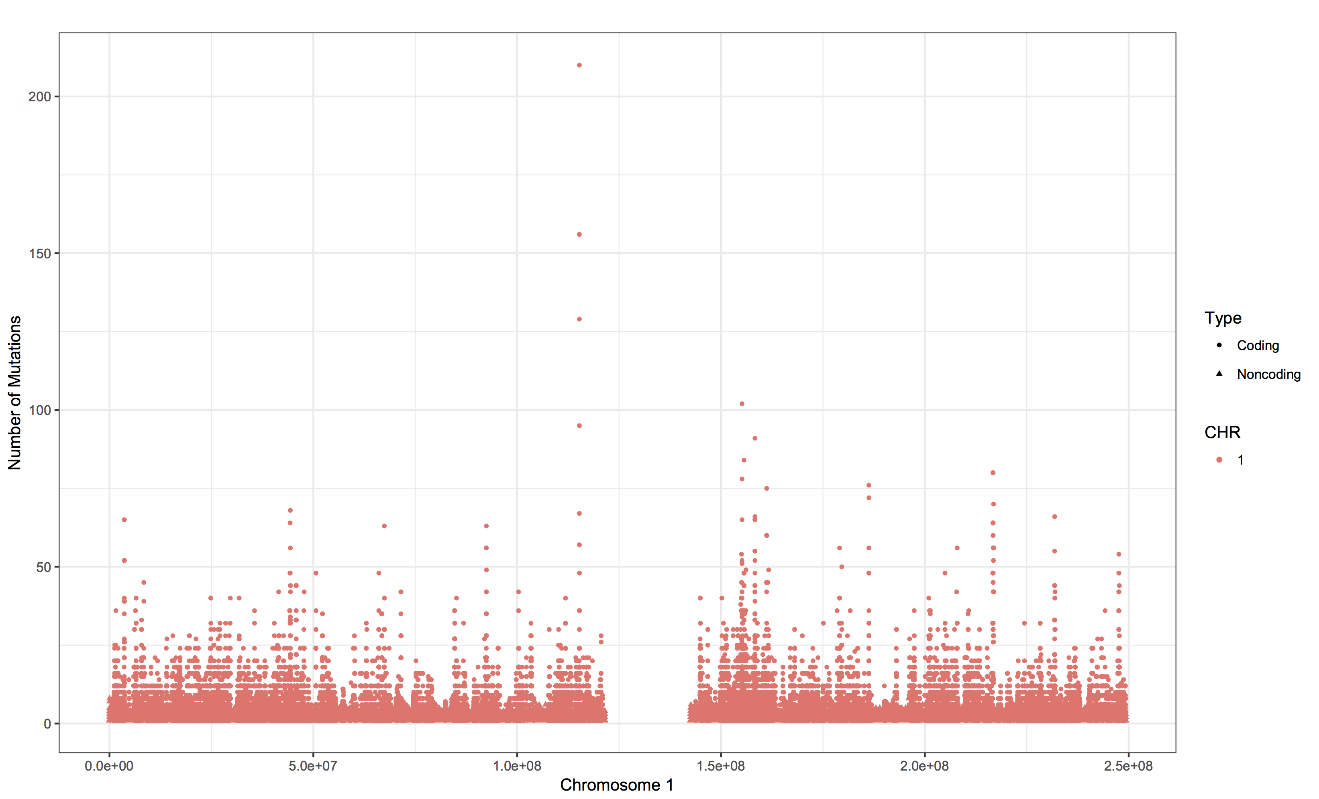


Figure S1-b: Distribution of SNVs in chromosome 2


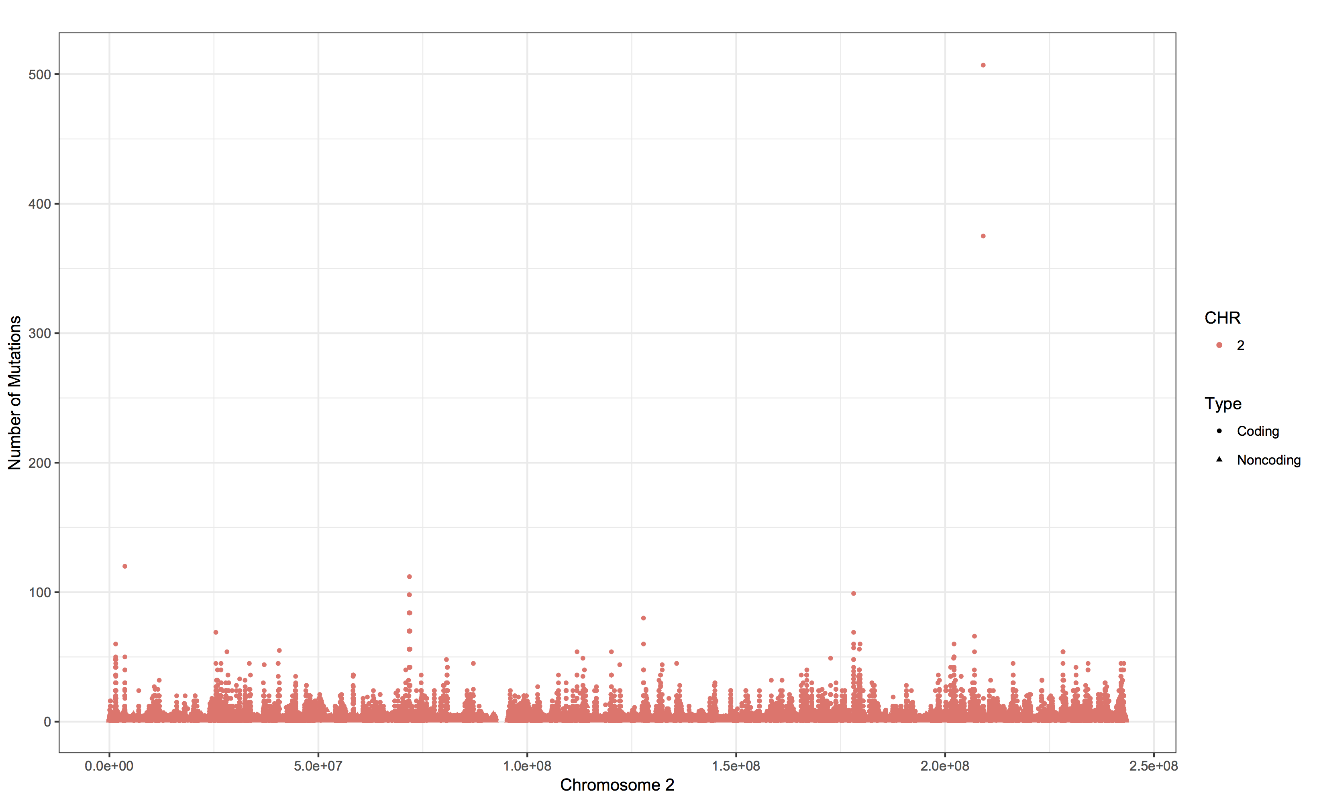


Figure S1-c: Distribution of SNVs in chromosome 3.


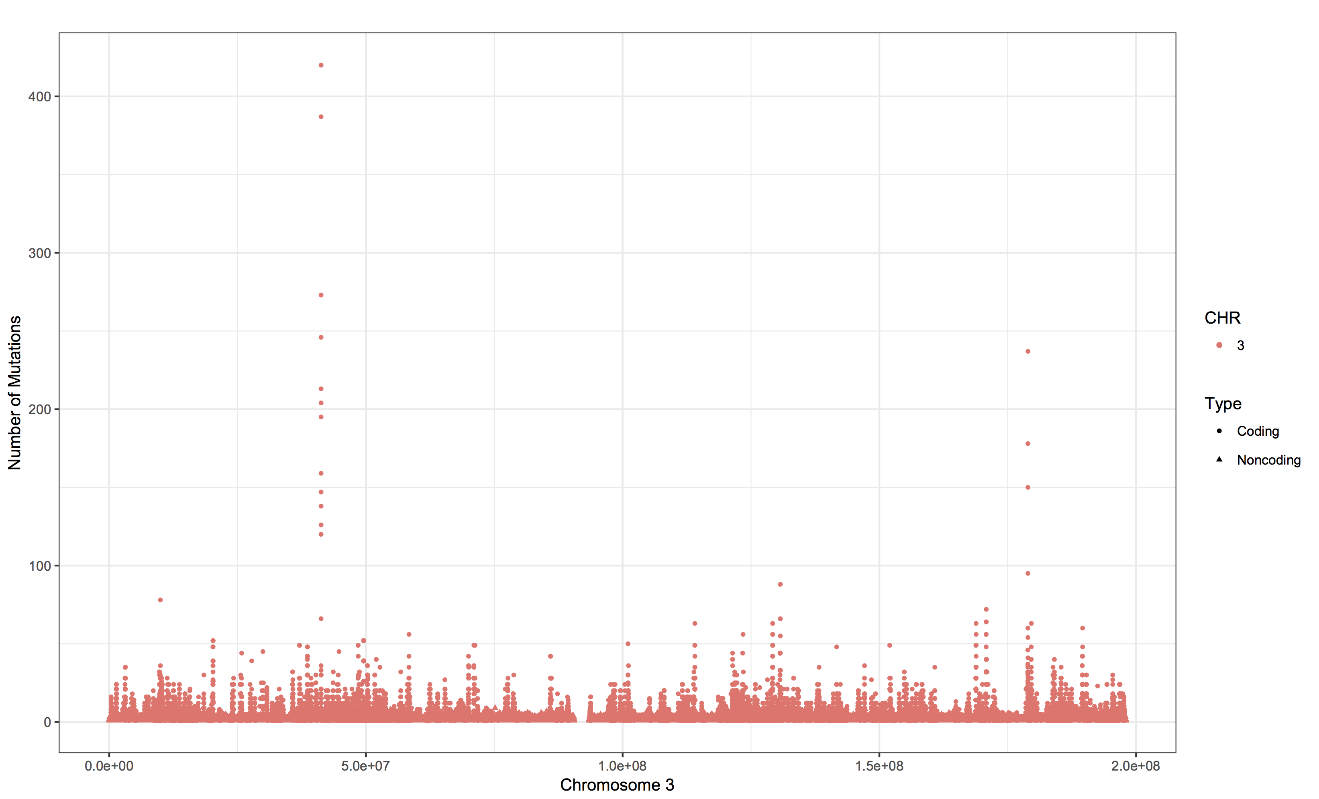


Figure S1-d: Distribution of SNVs in chromosome 4.


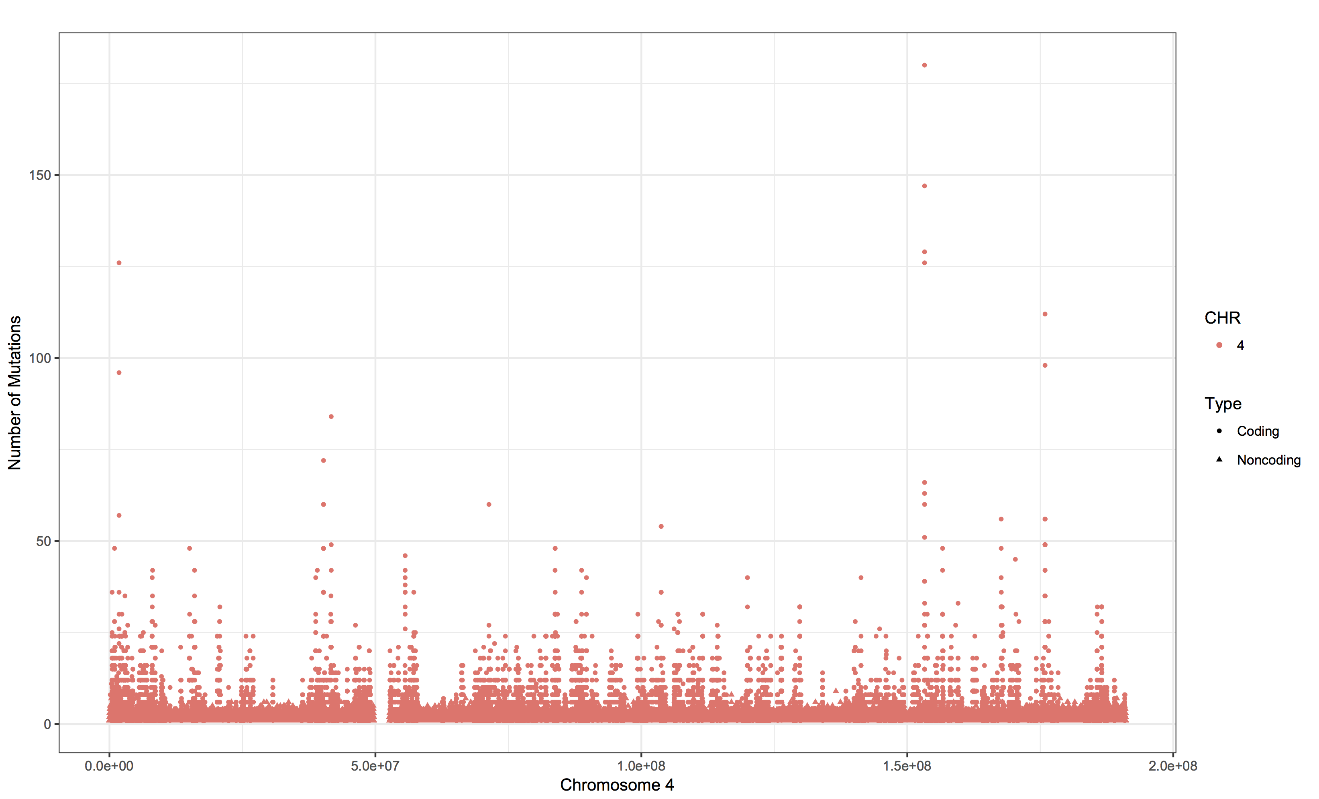


Figure S1-e: Distribution of SNVs in chromosome 5.


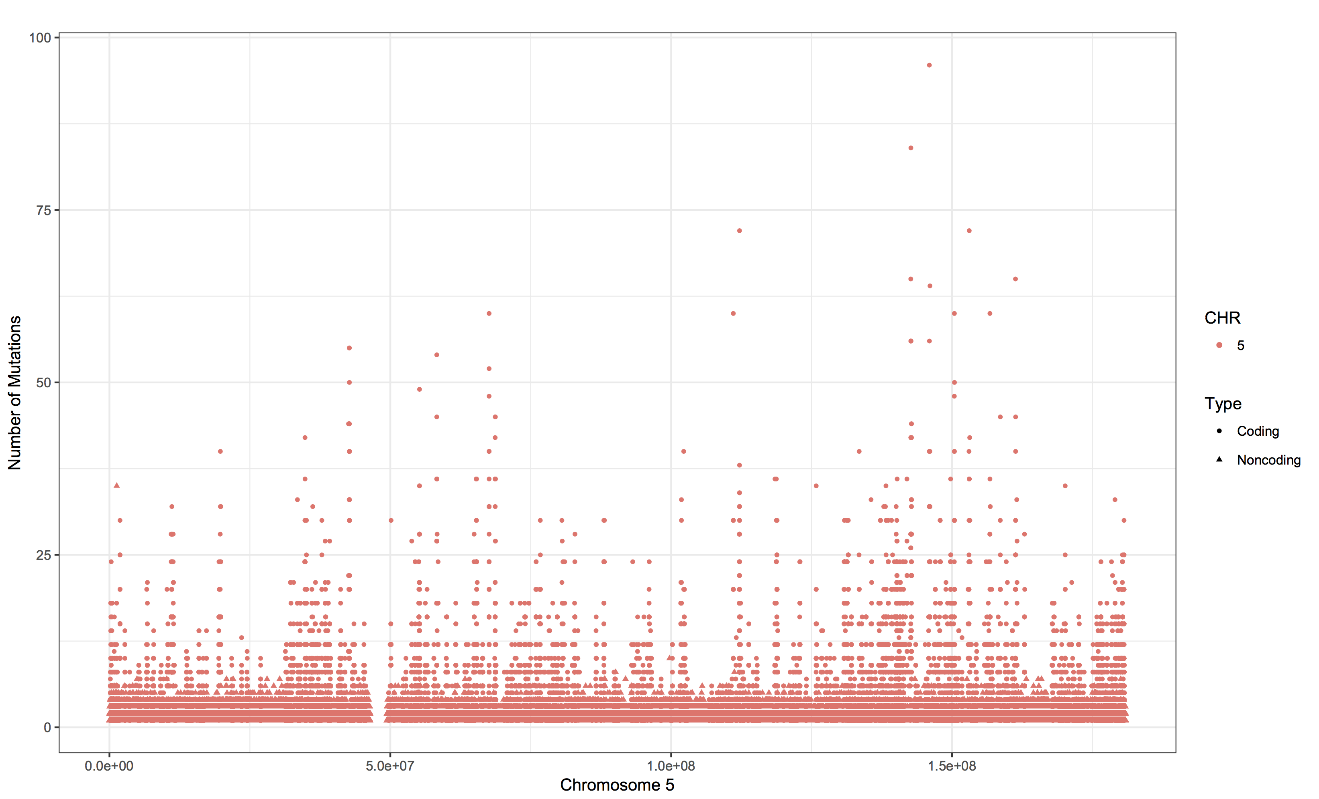


Figure S1-f: Distribution of SNVs in chromosome 6.


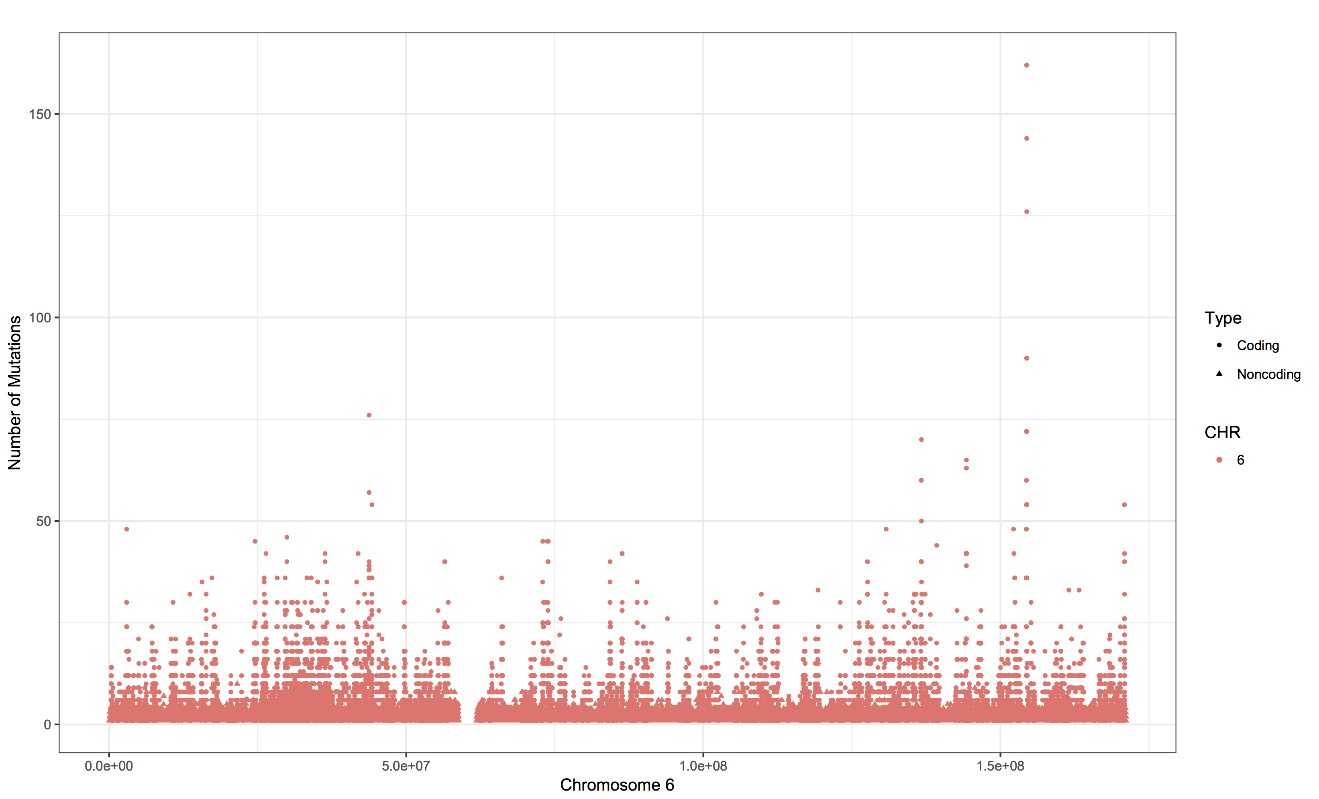


Figure S1-g: Distribution of SNVs in chromosome 7.


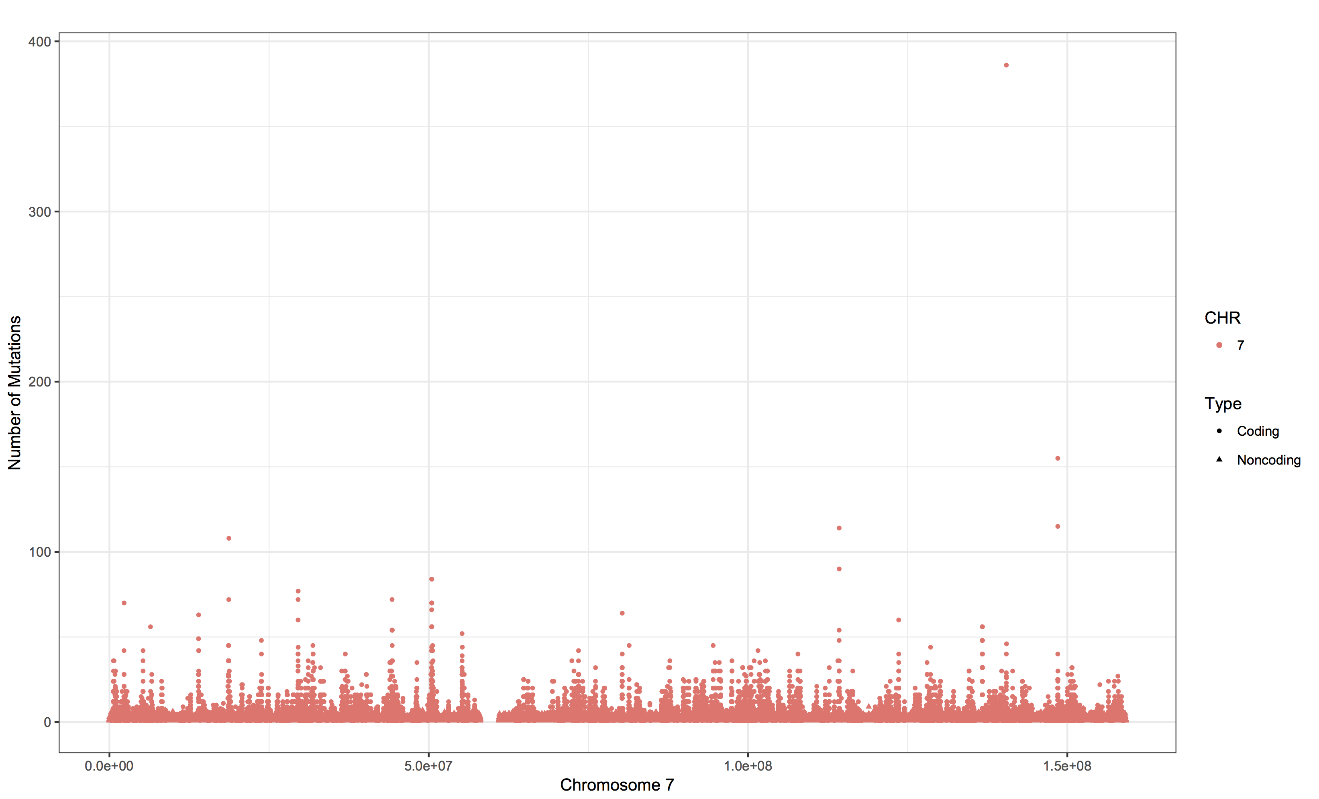


Figure S1-h Distribution of SNVs in chromosome 8.


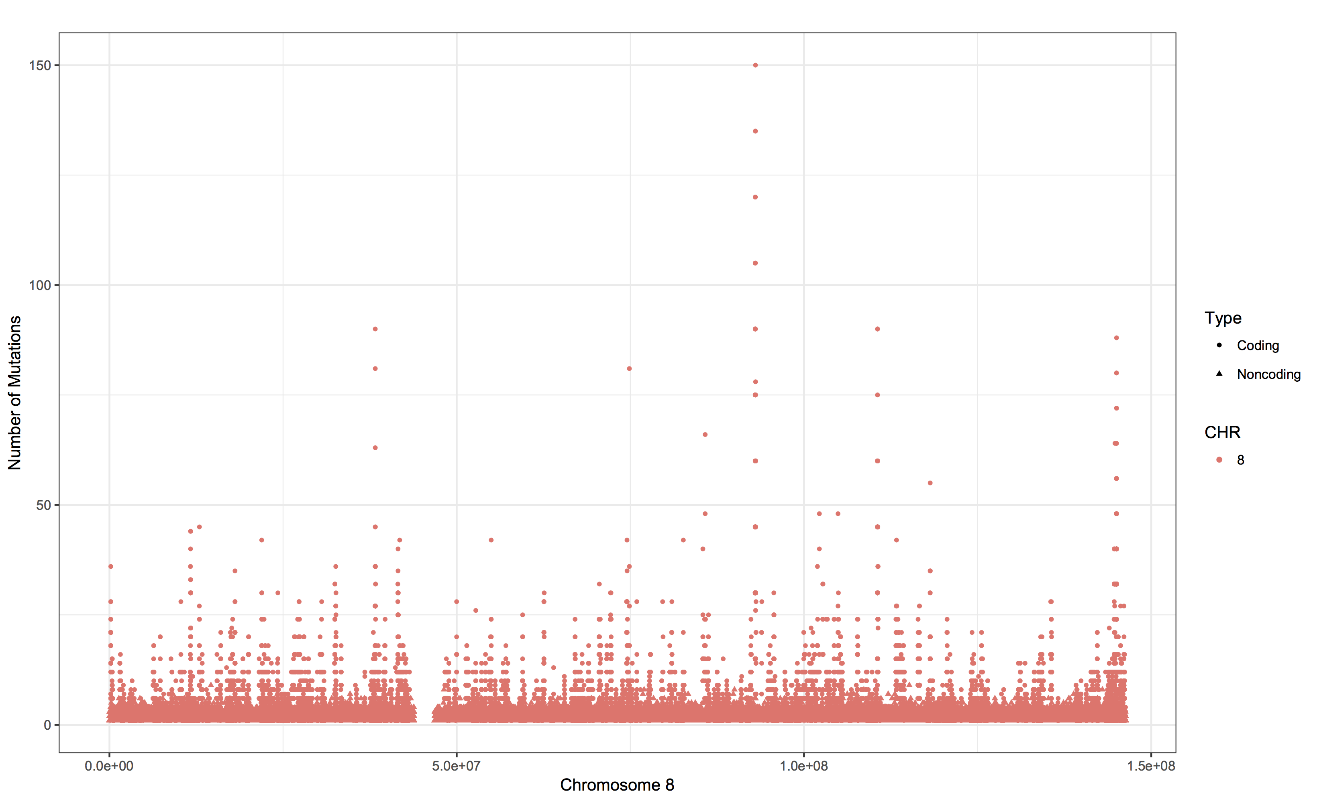


Figure S1-i: Distribution of SNVs in chromosome 9.


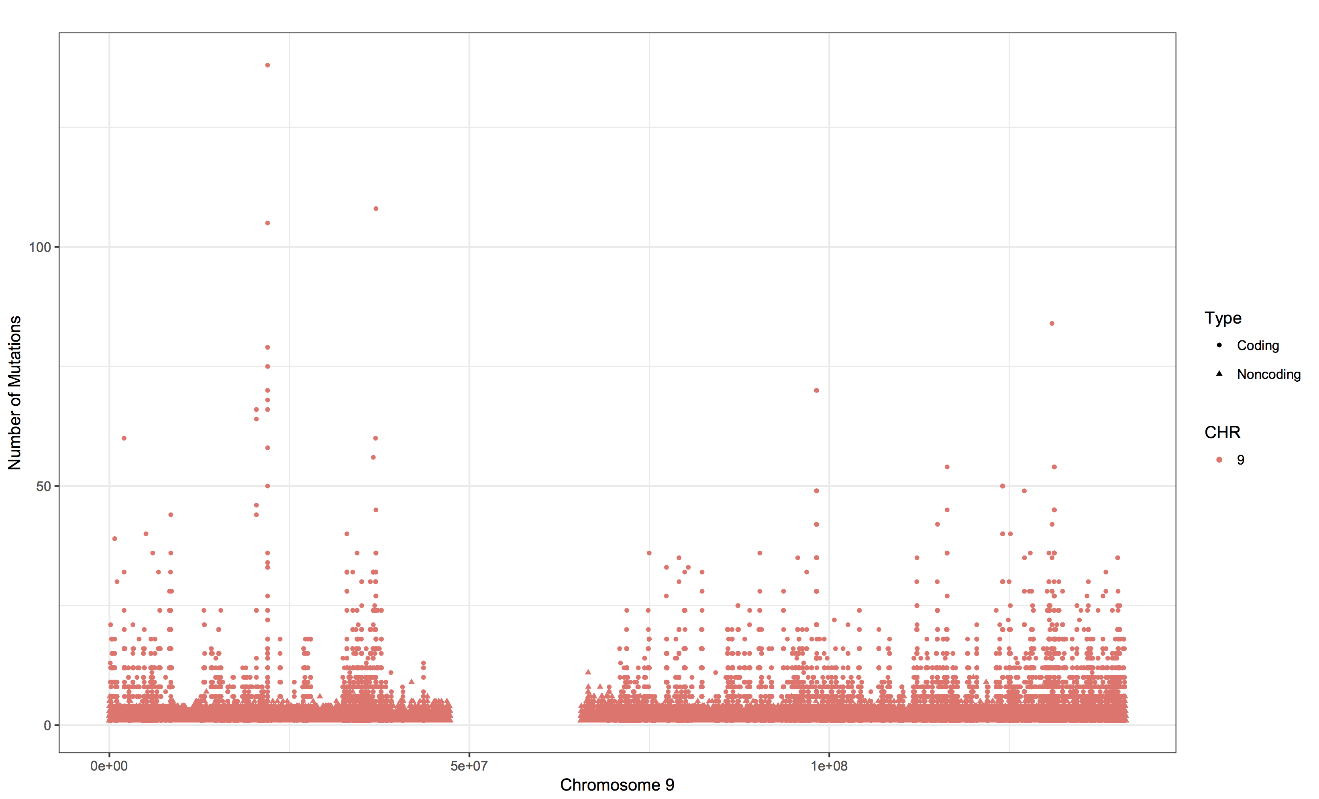


Figure S1-j: Distribution of SNVs in chromosome 10.


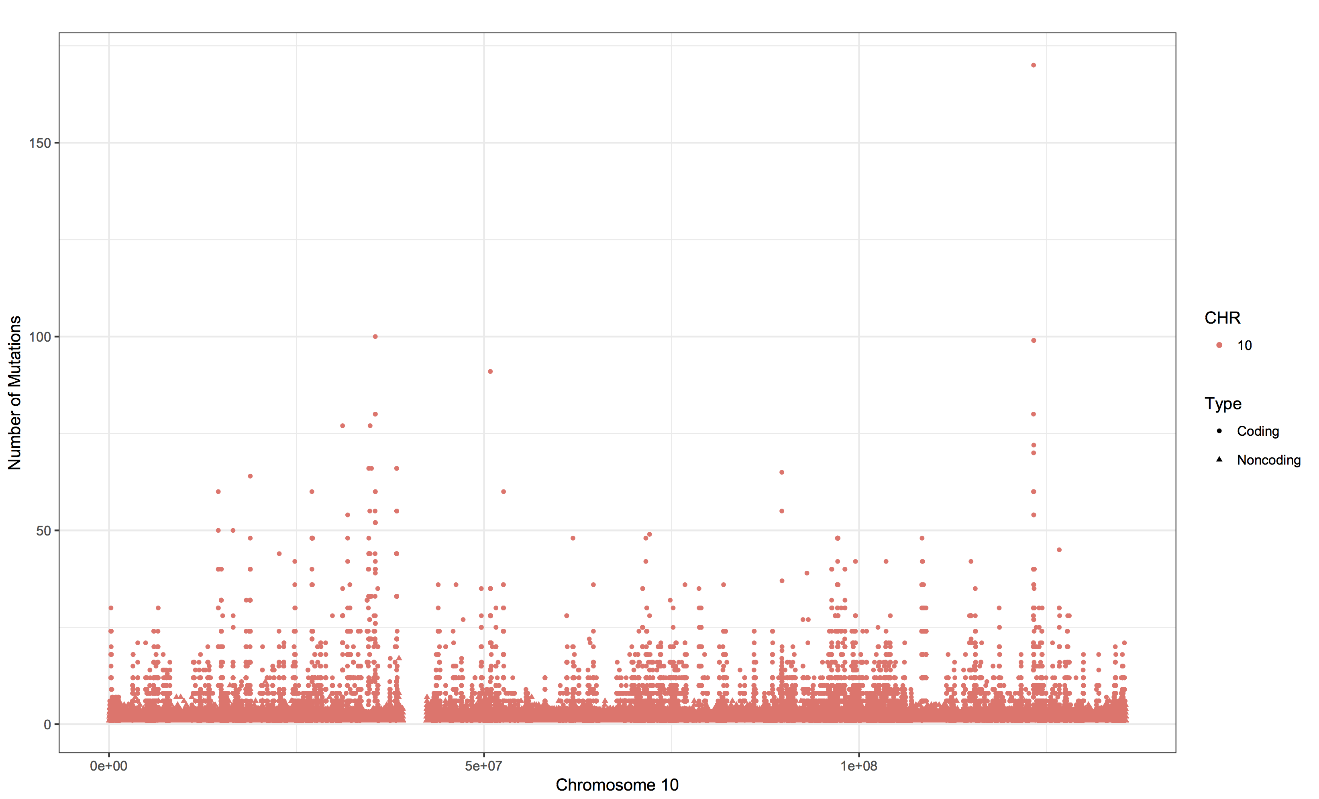


Figure S1-k: Distribution of SNVs in chromosome 11.


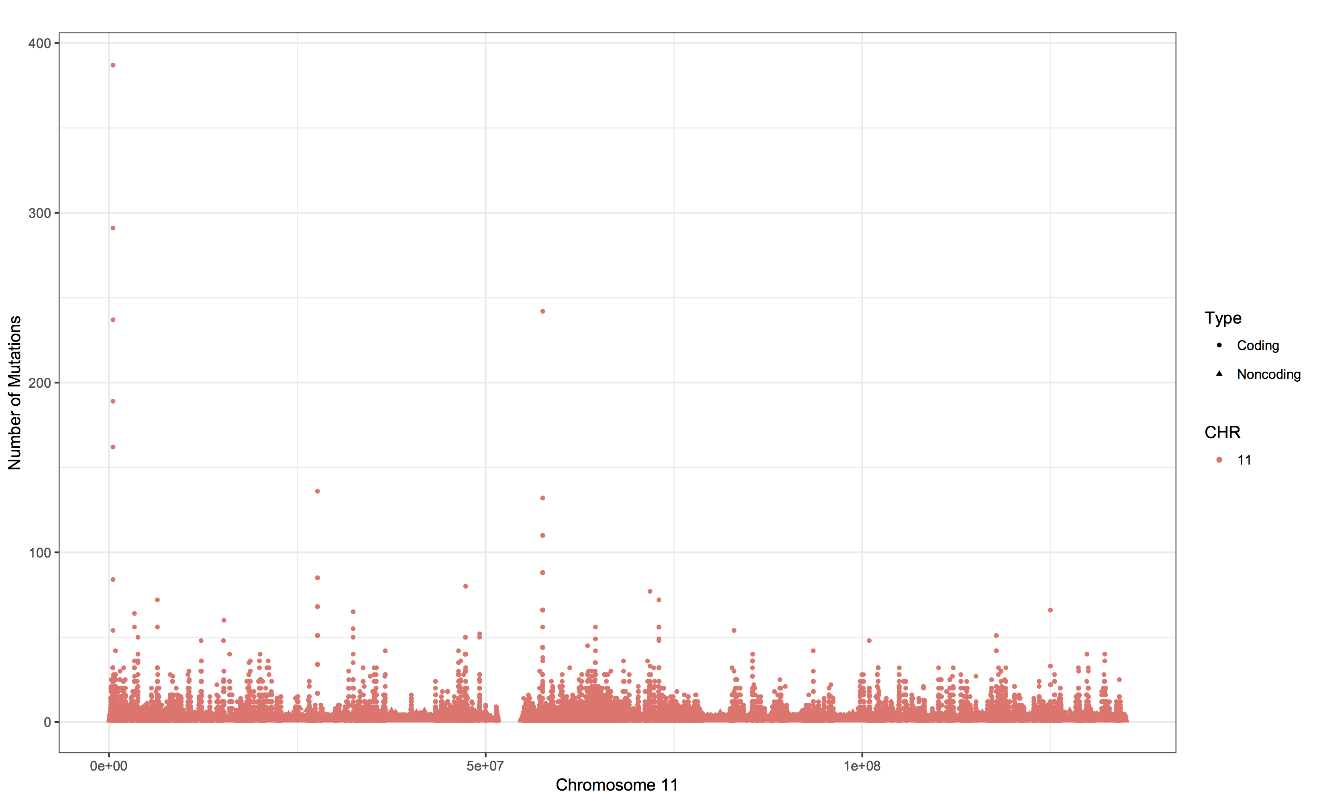


Figure S1-l: Distribution of SNVs in chromosome 12.


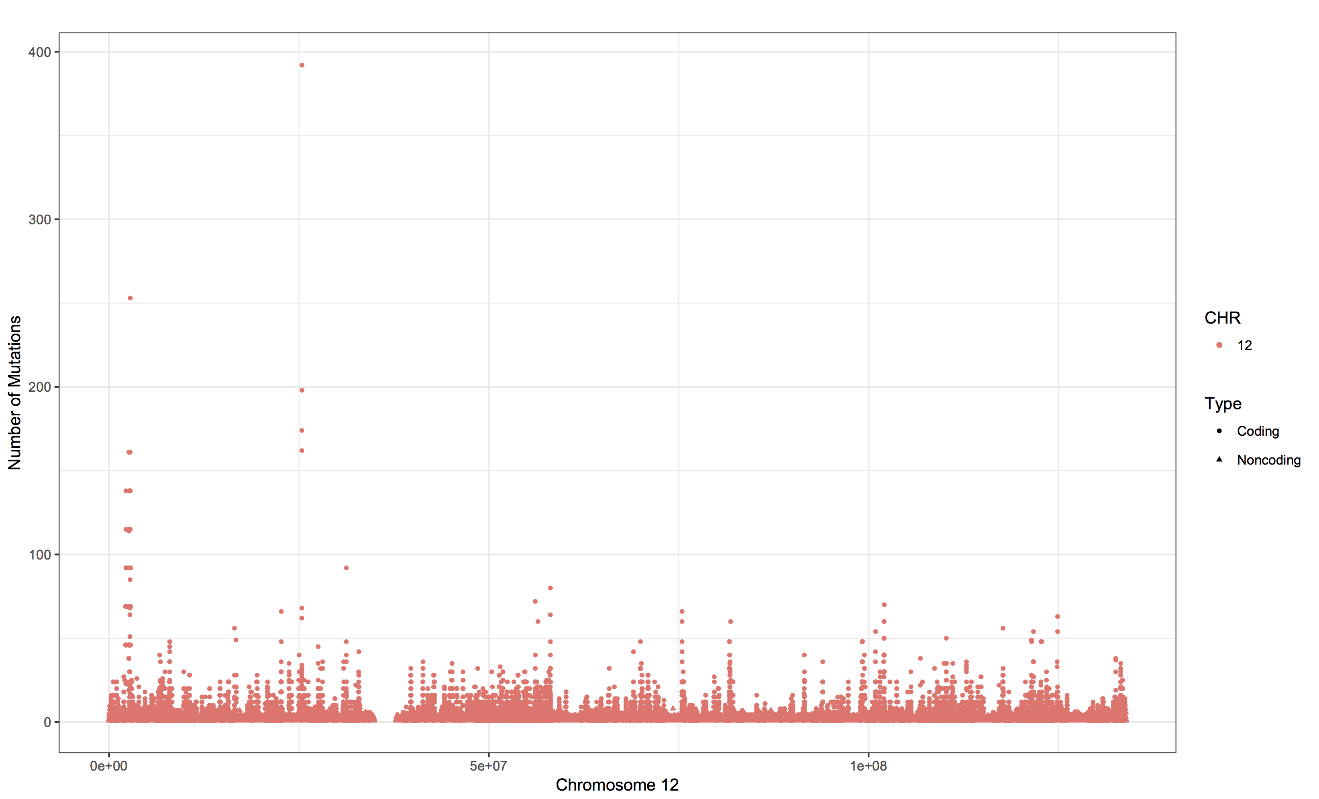


Figure S1-m: Distribution of SNVs in chromosome 13.


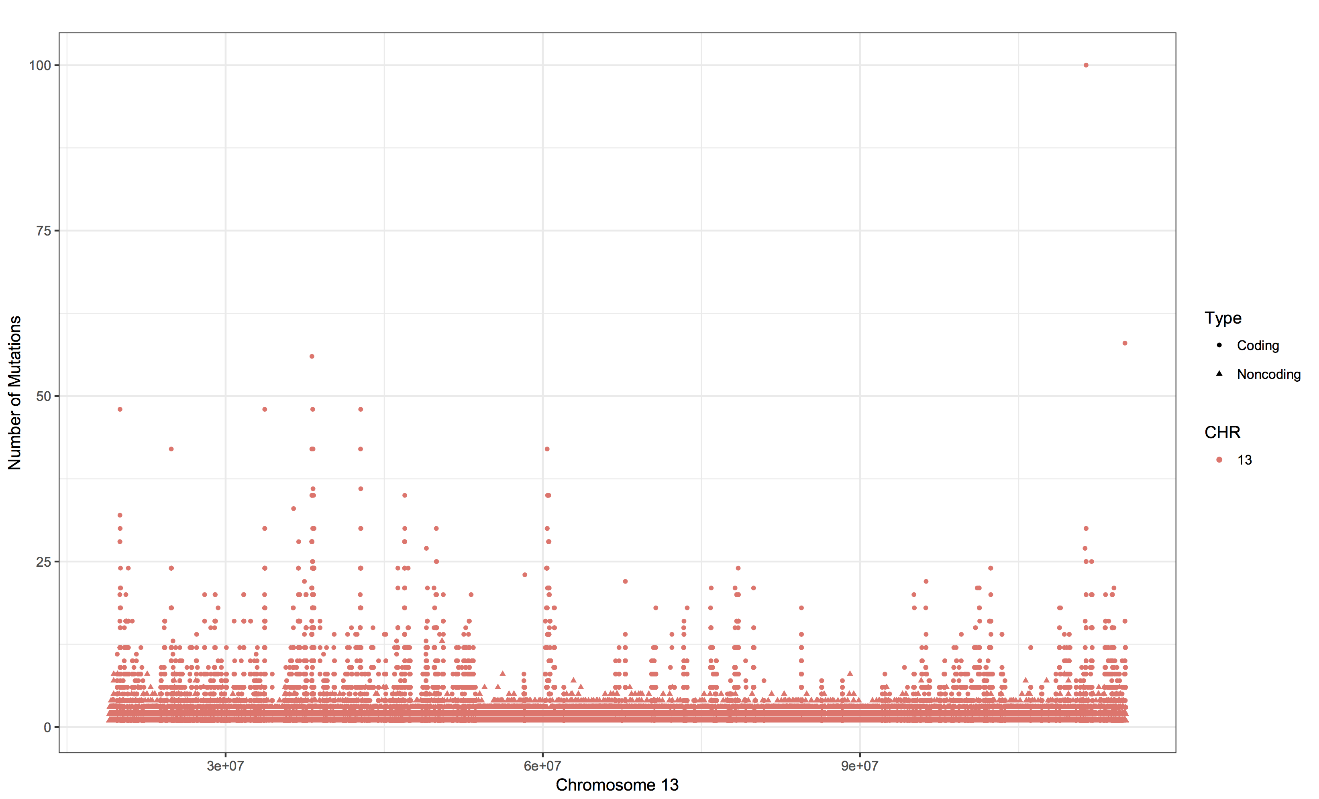


Figure S1-n: Distribution of SNVs in chromosome 14.


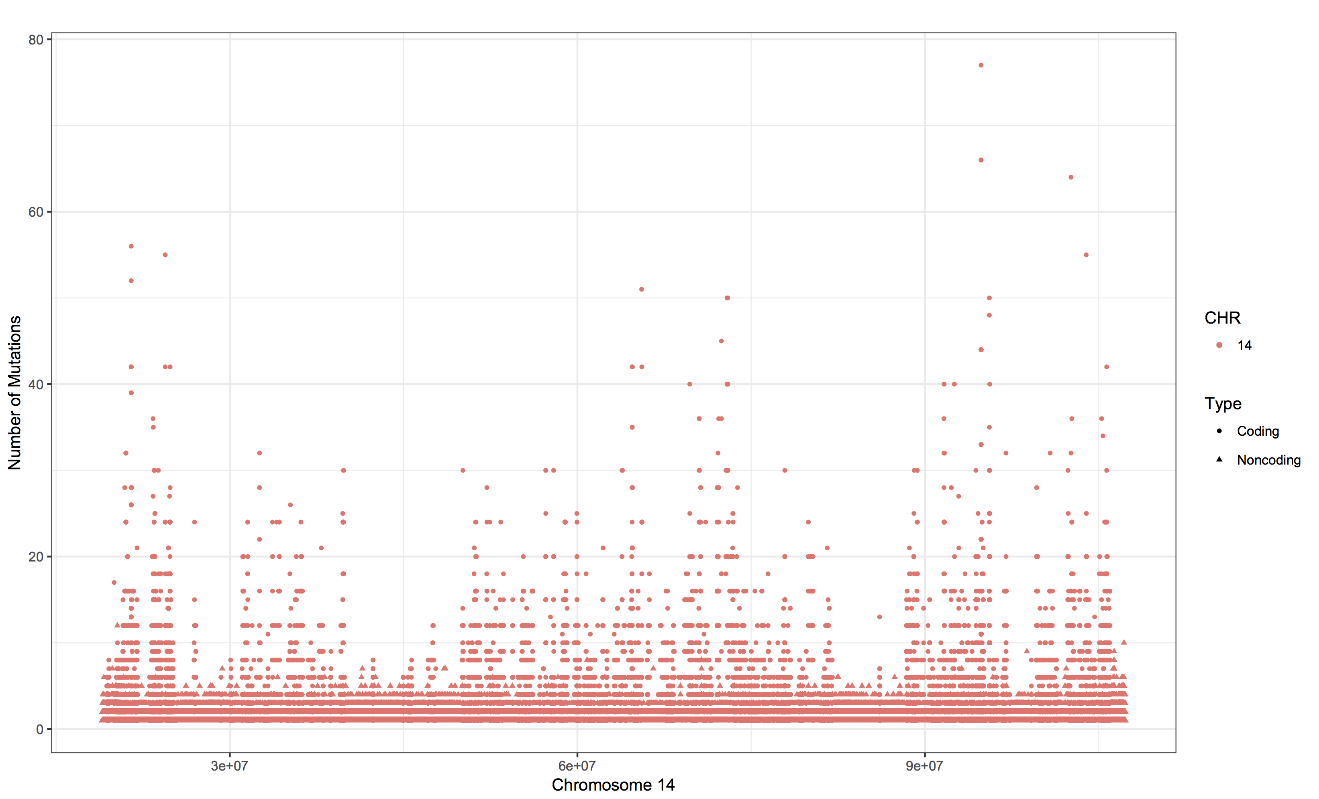


Figure S1-o: Distribution of SNVs in chromosome 15.


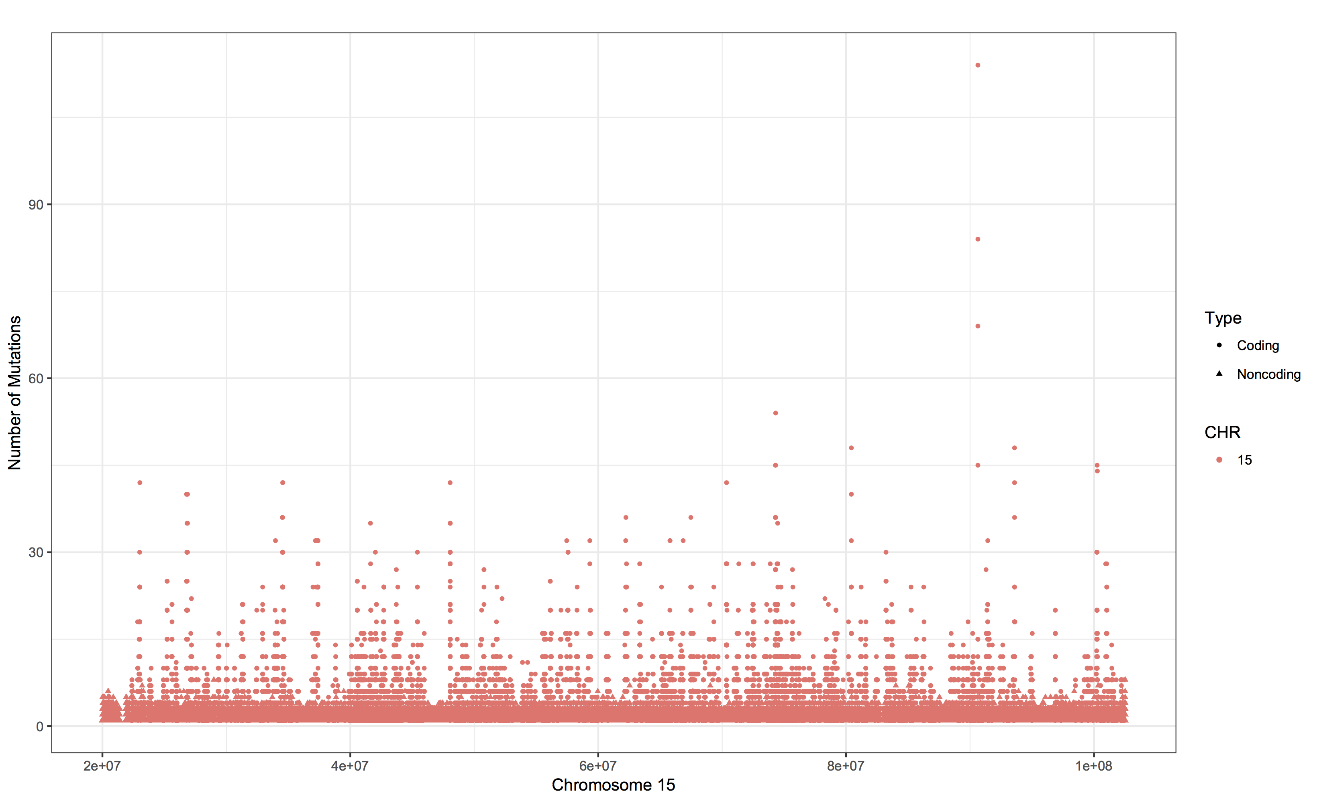


Figure S1-p: Distribution of SNVs in chromosome 16.


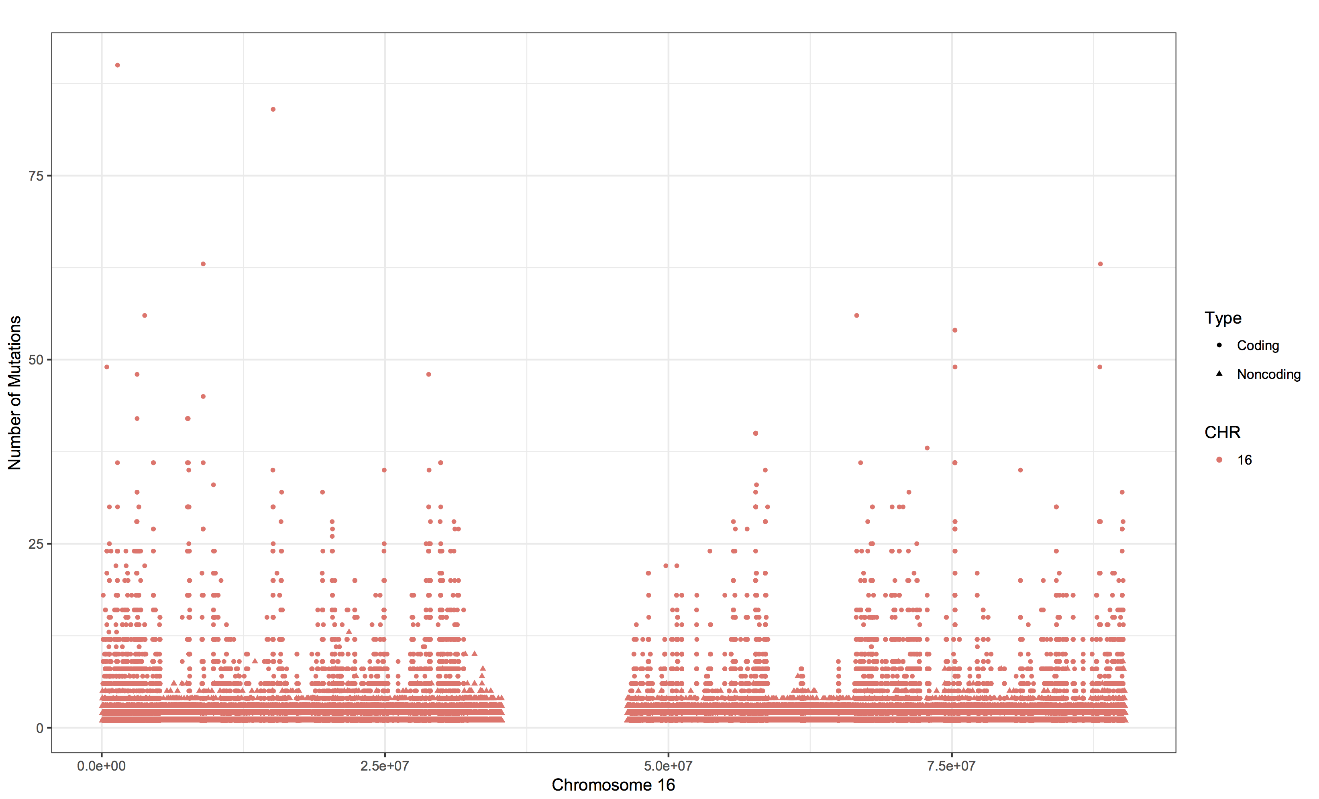


Figure S1-q: Distribution of SNVs in chromosome 17.


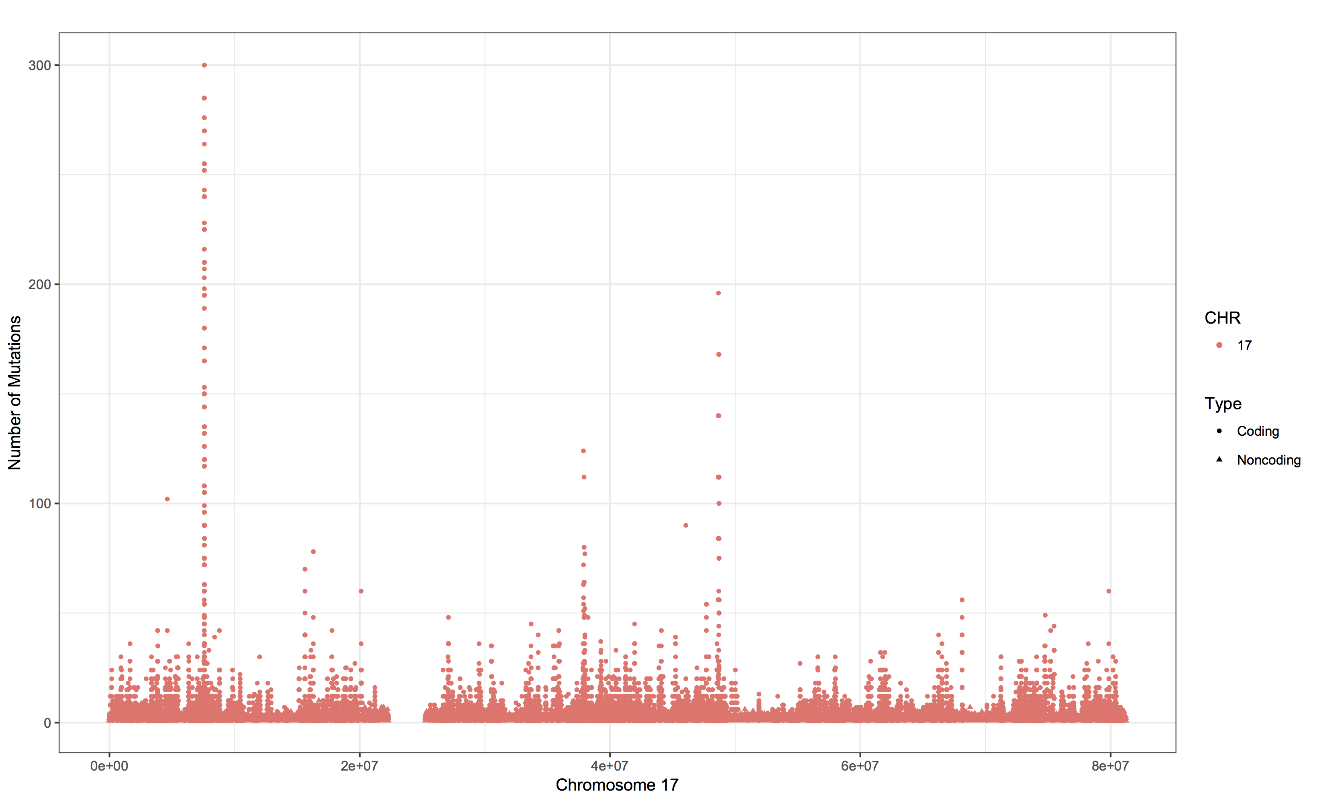


Figure S1-r: Distribution of SNVs in chromosome 18.


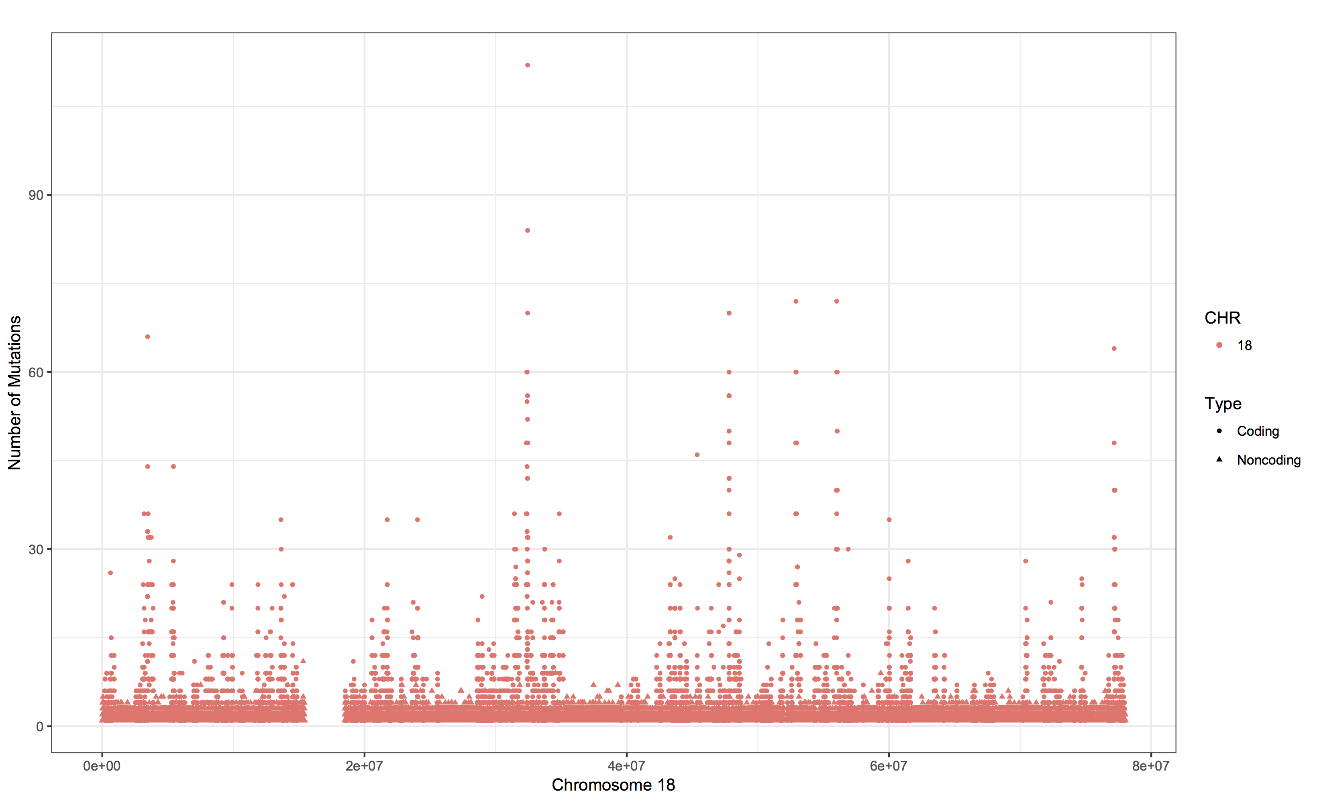


Figure S1-s: Distribution of SNVs in chromosome 19.


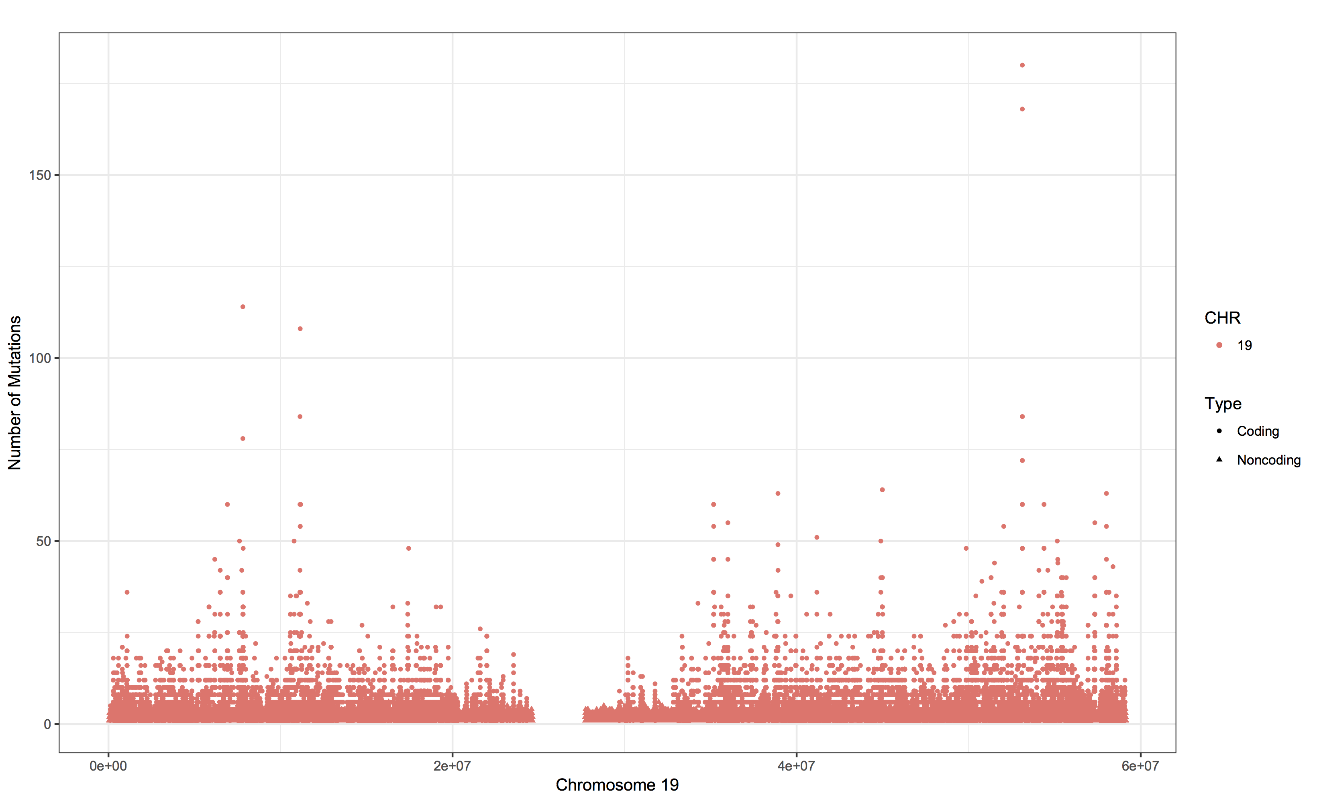


Figure S1-t: Distribution of SNVs in chromosome 20.


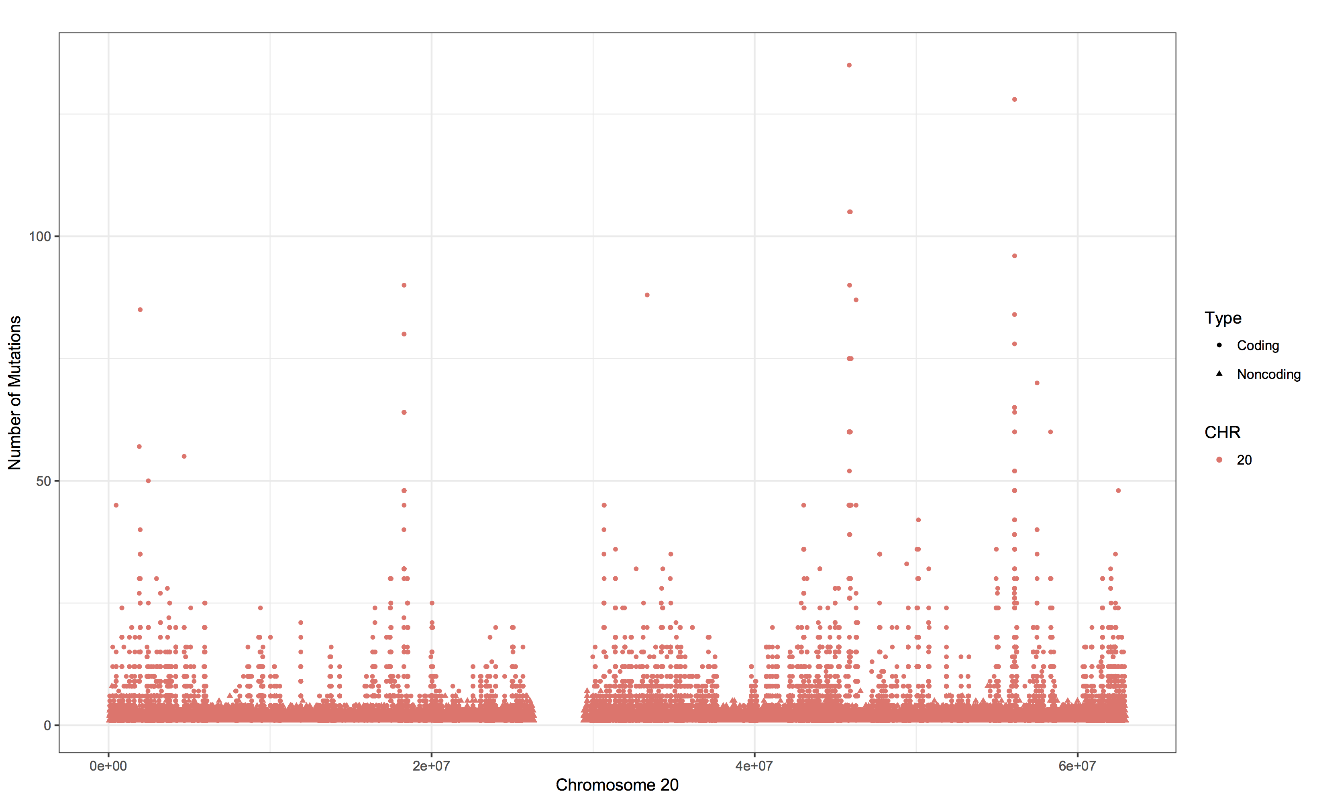


Figure S1-u: Distribution of SNVs in chromosome 21.


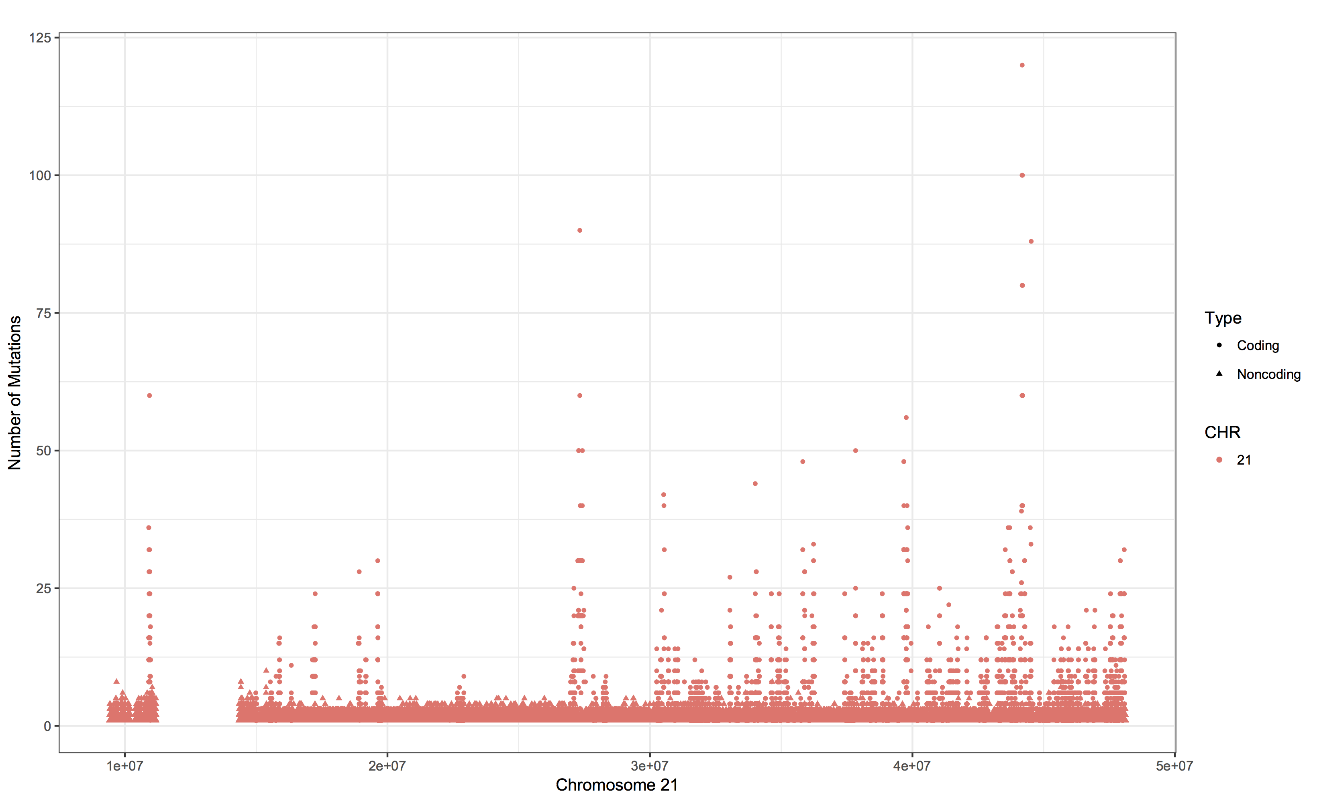


Figure S1-v: Distribution of SNVs in chromosome 22.


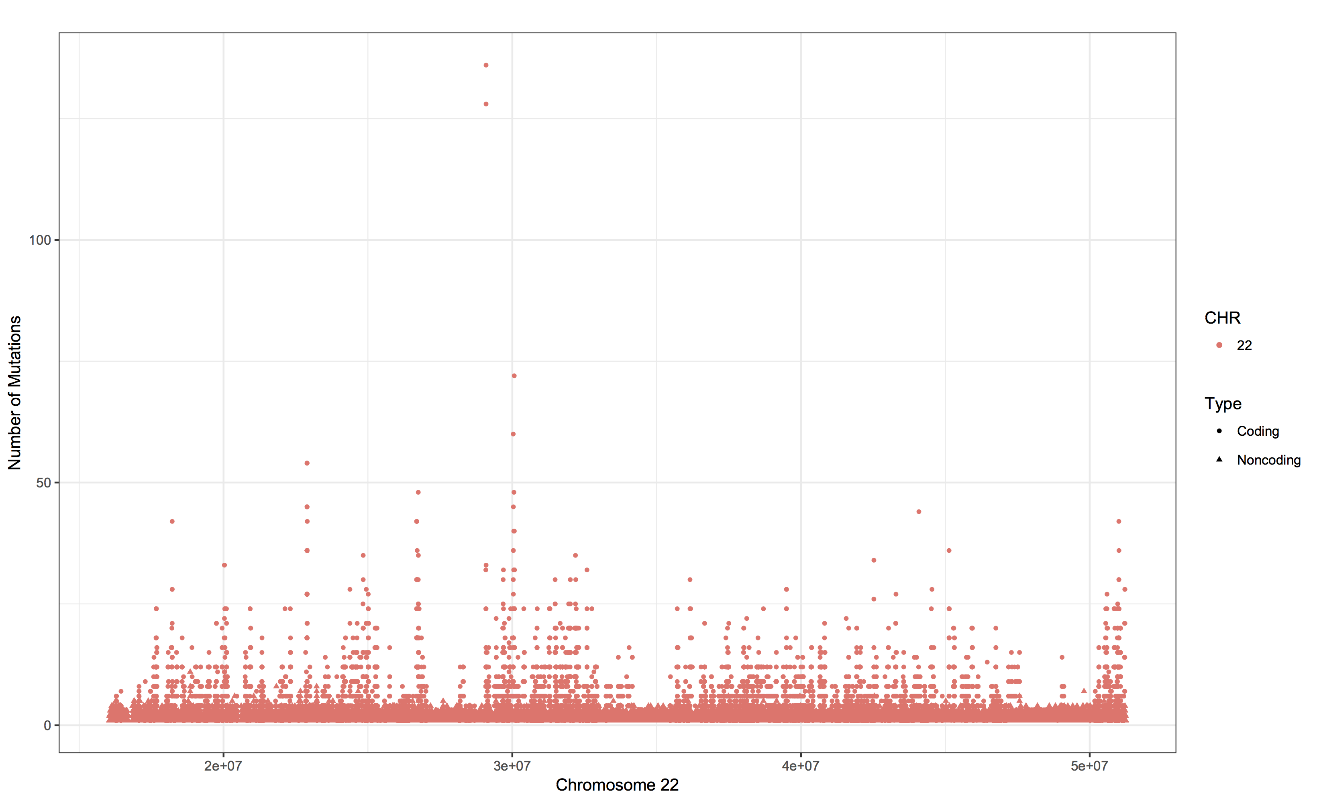


Figure S1-w: Distribution of SNVs in chromosome X.


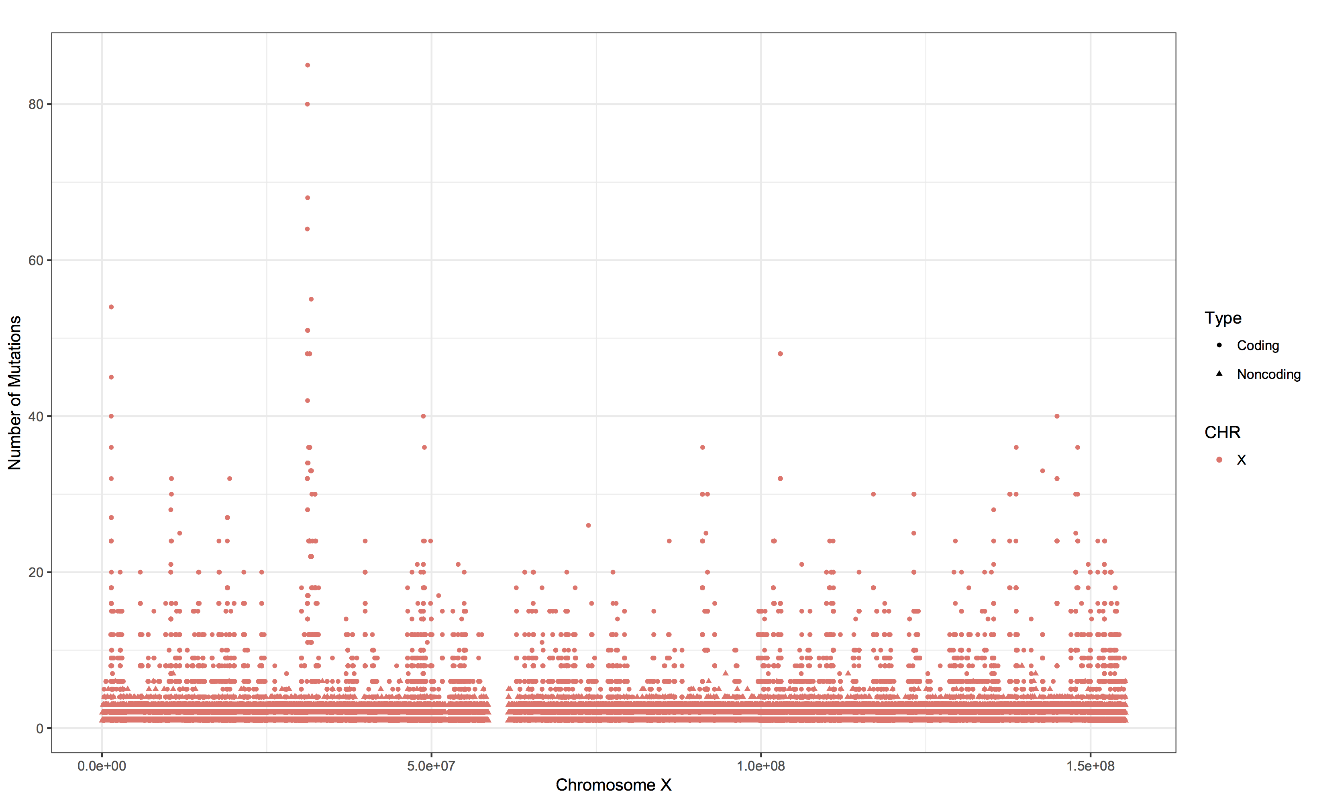


Figure S1-x: Distribution of SNVs in chromosome Distribution of SNVs in chromosome Y.


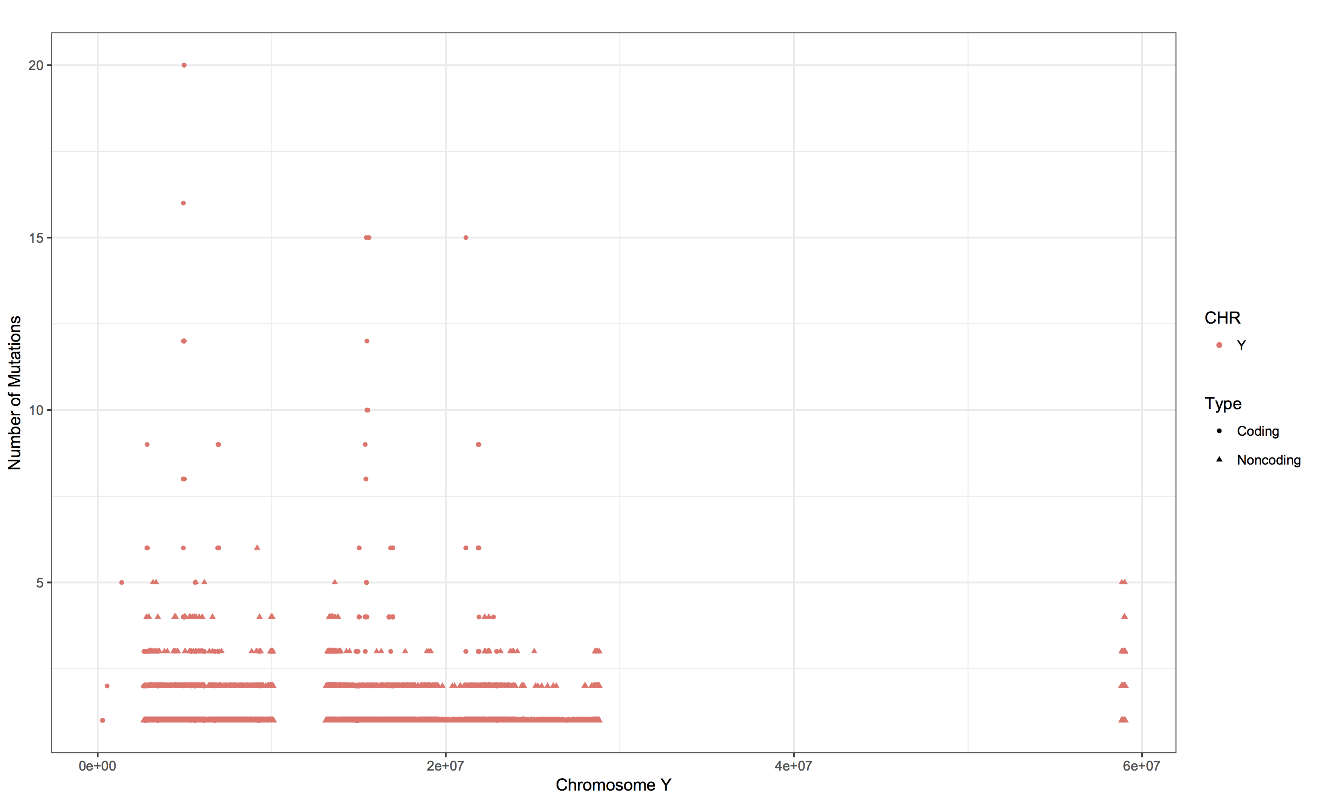

Supplement: S1 File — a~x The distribution of nsSNVs in protein coding region and SNVs in non-coding region by chromosome 1–22, X, Y. X-axis indicates the genomic positions of chromosome. Y-axis indicates the count of mutations. This Manhattan plot represents the distribution of mutations in all positions of the chromosome except for the centromere (empty region). (DOCX) [file pone.0213770.s001.docx]
