# Supplementary figures and images for: Investigation of somatic single nucleotide variations in human endogenous retrovirus elements and their potential association with cancer
Source: PLoS One. 2019 Apr 1;14(4):e0213770. doi: 10.1371/journal.pone.0213770 (PMC6443178; doi:10.1371/journal.pone.0213770)

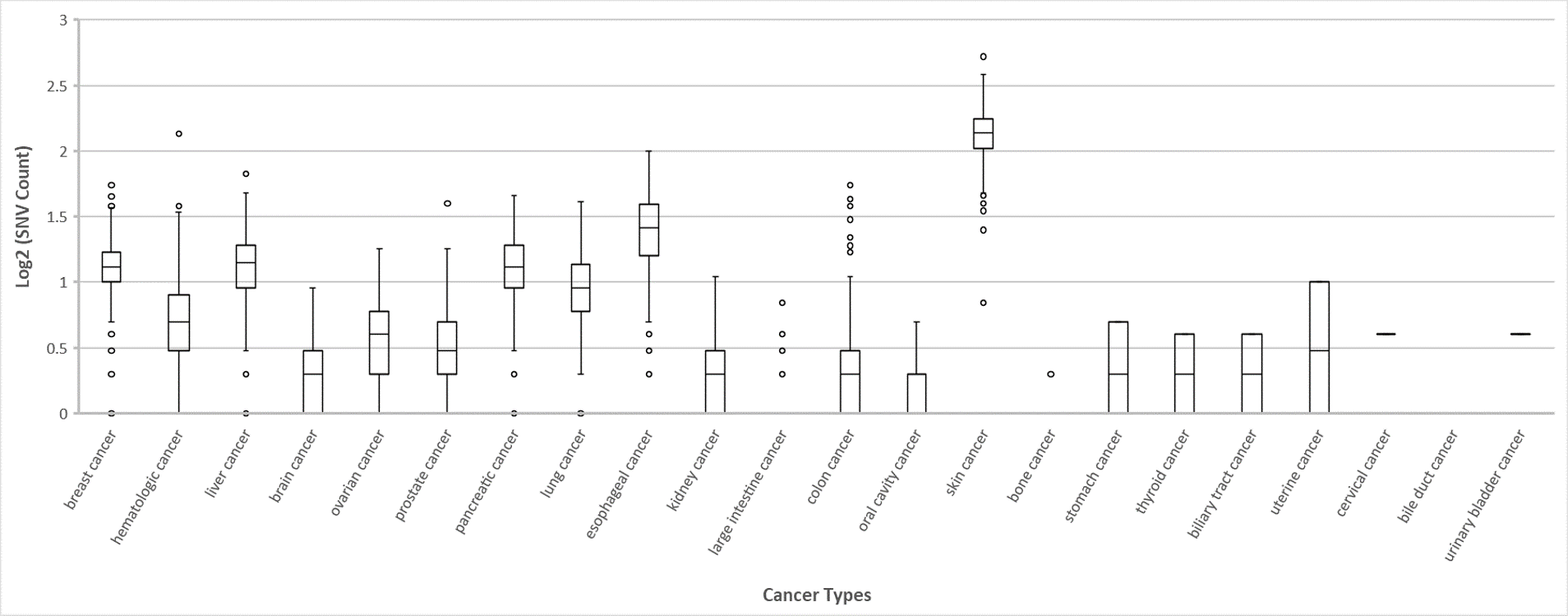

Supplement: S1 Fig — Each HERV element with count of somatic SNVs within non-coding region. X-axis indicates cancer types. Y-axis indicates Log2Counts (counts mean the number of SNVs). Box plot represents the number of SNVs located in the HERV elements. (TIFF) [file pone.0213770.s002.tiff]
